# Manganese Transport and Sediment Remobilization in a Tidal Estuary: Parameter Sensitivity and Uncertainty Analysis

**DOI:** 10.3390/toxics14100916

**Published:** 2026-10-16

**Authors:** Hong-Ming Liu, Wen-Cheng Liu, Wei-Che Huang, Chih-Chieh Young

**Affiliations:** 1 Department of Civil and Disaster Prevention Engineering, National United University, Miaoli 360302, Taiwan; dslhmd@gmail.com; 2 Department of Marine Environmental Informatics, National Taiwan Ocean University, Keelung 20224, Taiwan; youngjay@ntou.edu.tw

**Keywords:** heavy metal–manganese, tidal estuary, uncertainty analysis, Monte Carlo simulation, SCHISM-HM

## Abstract

Tidal estuaries are characterized by complex hydrodynamic processes, while heavy metal transport is governed by multiple interacting physical mechanisms. These complexities introduce uncertainties in key model parameters and may compromise the reliability of model predictions. This study developed a three-dimensional hydrodynamic–heavy metal transport model (SCHISM-HM) for tidal estuaries and applied it to the Danshuei River estuarine system in Taiwan to systematically investigate manganese transport mechanisms, parameter sensitivity, and prediction uncertainty. The model was validated against field observations collected in 2020. The simulated hydrodynamic variables, including water level, velocity, and salinity, showed good agreement with observations, with skill scores (*SS*) exceeding 0.8. For total manganese concentrations, the mean absolute error (*MAE*) ranged from 0.001 to 0.006 mg/L, the root mean square error (*RMSE*) from 0.001 to 0.008 mg/L, and the *SS* from 0.909 to 0.999, demonstrating the capability of SCHISM-HM to reproduce the complex hydrodynamic conditions and heavy metal transport processes in the Danshuei River estuary. Sensitivity analysis revealed that both the partition coefficient (*K_d_*) and suspended sediment settling velocity (*w_s_*) were negatively correlated with total manganese concentrations in the water column. Increasing *K_d_* from 0.1 to 0.4 L/mg resulted in reductions in peak total manganese concentrations of 32.03–93.75% across the monitoring stations. Conversely, decreasing *w_s_* from 7.0 to 3.0 m/day increased total manganese concentrations at downstream stations by up to 382.08%. These results indicate that *w_s_* exerts a stronger influence on total manganese concentrations than *K_d_*, highlighting the critical role of suspended sediment transport in controlling heavy metal distributions. Model uncertainty was further quantified by integrating Monte Carlo simulation with Bayesian inference. The posterior distributions of both *K_d_* and *w_s_* were best represented by normal distributions, with correlation coefficients (*R*) exceeding 0.997. Uncertainty in simulated total manganese concentrations was predominantly associated with peak-concentration periods and exhibited pronounced spatial variability. In particular, the Taipei Bridge station, where river cross-sectional contraction enhances hydrodynamic forcing and sediment remobilization, exhibited the largest 90% confidence interval width (0.118 mg/L) and a relatively high probability of exceeding the water quality standard. Overall, the results demonstrate that bed sediment erosion and suspended sediment transport are key processes governing manganese dynamics in the Danshuei River estuary. The proposed modeling and uncertainty-analysis framework provides a quantitative basis for water pollution management and environmental risk assessment in tidal estuaries.

## 1. Introduction

Continuous industrialization and urbanization have progressively intensified the contamination of natural resources. Among various pollutants, heavy metals have long been recognized as a critical global environmental issue. Heavy metals, typically defined as metallic elements with relatively high densities, pose substantial ecological and human-health risks due to their persistence in aquatic environments. These substances do not readily degrade and can accumulate in aquatic organisms, resulting in bioaccumulation and biomagnification throughout the food web. Consequently, heavy metal concentrations tend to increase across trophic levels, ultimately threatening human health through dietary exposure, where excessive intake may disrupt metabolic processes, impair cellular functions, and in severe cases cause irreversible neurological damage. For these reasons, heavy metal concentrations are widely adopted as key indicators for water quality assessment [1,2,3]. Historical pollution events across Asia, such as mercury-induced Minamata disease and cadmium-related itai-itai disease in Japan [4,5], and arsenic-associated blackfoot disease and copper-related green oyster contamination in Taiwan [6,7], further demonstrate the profound hazards posed by heavy metals to public health.

Sources of heavy metals in aquatic systems can be broadly categorized as natural or anthropogenic. Natural inputs originate from trace metals in the Earth’s crust released through rock weathering and geological erosion, forming background concentrations in water bodies. In contrast, anthropogenic inputs arise from mining, smelting, industrial effluents, municipal wastewater, and agricultural runoff, all of which substantially elevate metal concentrations in aquatic environments [8,9,10]. In addition to sources, metal speciation governs their environmental behavior. Heavy metals occur mainly in dissolved or particulate-bound forms, with the latter typically adsorbed onto suspended sediments or associated with colloids and fine particles. Suspended sediment serves as the principal carrier of particulate-bound metals. Under weak hydrodynamic conditions, sediment-associated metals tend to settle onto the bed, forming long-term reservoirs. When hydrodynamic energy increases and resuspends bed material, previously deposited metals may be released back into the water column, potentially causing secondary contamination [11,12].

Approaches for assessing heavy metal concentrations in aquatic systems generally fall into three categories: field monitoring, statistical analysis, and numerical modeling. Field monitoring offers direct observations of spatiotemporal variability, yet it is often constrained by limited sampling frequency, spatial coverage, manpower, and cost [13]. Statistical methods help identify distribution patterns, correlations, and dominant influencing factors, but their effectiveness is restricted by data availability and their inability to fully capture the complex physical, chemical, and biological processes governing metal transport [14,15]. Numerical modeling provides a complementary and integrative approach by coupling hydrodynamics, suspended sediment transport, and heavy metal transport processes to simulate exchanges among the water column, suspended particulates, and bed sediments. This approach has been widely applied to reproduce spatiotemporal metal dynamics across diverse aquatic environments [16,17,18]. Numerical modeling not only compensates for the limitations of field- and data-based approaches but also facilitates scenario prediction under varying hydrological and environmental conditions, supporting risk assessment and environmental management.

In numerical simulations of heavy metal transport, the specification of key parameters strongly influences model performance. Among these parameters, the partition coefficient, which governs the distribution of metals between dissolved and particulate phases, is particularly critical, as it directly affects metal migration and dispersion. Numerous studies have shown that partition coefficients vary widely depending on water quality characteristics, suspended sediment properties, and metal species [19,20,21,22]. However, because these parameters are often derived from literature values or limited field measurements, they inevitably contain uncertainties. Such uncertainties can propagate through simulations, influencing predicted spatial patterns of heavy metal concentrations and potentially biasing pollution source identification and risk assessments. Monte Carlo simulation (MCS) is therefore commonly used to quantify parameter uncertainty and its impact on model predictions [23,24].

Compared with previous heavy-metal transport studies, this study integrates three-dimensional hydrodynamic–heavy metal transport modeling with parameter sensitivity analysis and probabilistic uncertainty assessment in a unified framework. In particular, the effects of key sediment-related parameters on manganese (Mn) transport are systematically evaluated, while their uncertainties are further quantified through MCS and Bayesian inference. This integrated approach provides a more comprehensive assessment of parameter influence and prediction uncertainty for heavy metal transport in tidal estuarine environments.

Despite the widespread application of MCS in environmental modeling, studies that explicitly address parameter uncertainty in heavy metal transport simulations remain limited, particularly those focusing on the partition coefficient. Accordingly, the objective of this study is to quantify the sensitivity and uncertainty of key heavy metal transport parameters, especially the partition coefficient in tidal estuarine environments. To achieve this, a three-dimensional hydrodynamic–heavy metal transport model tailored to estuarine conditions is first developed to characterize hydrodynamics and the spatiotemporal distribution of heavy metal concentrations. Parameter ranges are then established based on relevant literature and incorporated into an MCS framework for probabilistic sampling. Finally, multiple model realizations are conducted to evaluate how parameter uncertainties propagate and affect simulated heavy metal concentrations.

## 2. Description of Study Area and Data Collection

### 2.1. Study Area

The Danshuei River basin, located in northern Taiwan, was selected as the study area because it is the largest tidal river system in Taiwan. The basin covers approximately 2726 km^2^, representing about 7.6% of the island’s total land area, and encompasses parts of Taipei City, New Taipei City, Keelung City, Taoyuan City, and Hsinchu County. The Danshuei River originates from Pintian Mountain in the Central Mountain Range at an elevation of 3529 m and has a total channel length of approximately 158.7 km. The river system consists of three major tributaries: the Dahan, Xindian, and Keelung rivers. The Dahan and Xindian rivers converge at Jiangzicui to form the main stem of the Danshuei River, which subsequently joins the Keelung River at Guandu before discharging northwestward into the Taiwan Strait at Danshuei [25].

The mean discharges of the Dahan, Xindian, and Keelung rivers are 59.62, 92.27, and 39.50 m^3^/s, respectively. Hydrodynamics in the lower reaches are dominated by semidiurnal tides, primarily the M_2_ and S_2_ constituents, resulting in two flood tides and two ebb tides each day. The mean tidal range is approximately 2.22 m, increasing to about 3.10 m during spring tides [26,27]. Tidal influences extend upstream to Chenglin Bridge on the Dahan River, Xiulang Bridge on the Xindian River, and Jiangbei Bridge on the Keelung River.

**Figure 1:**
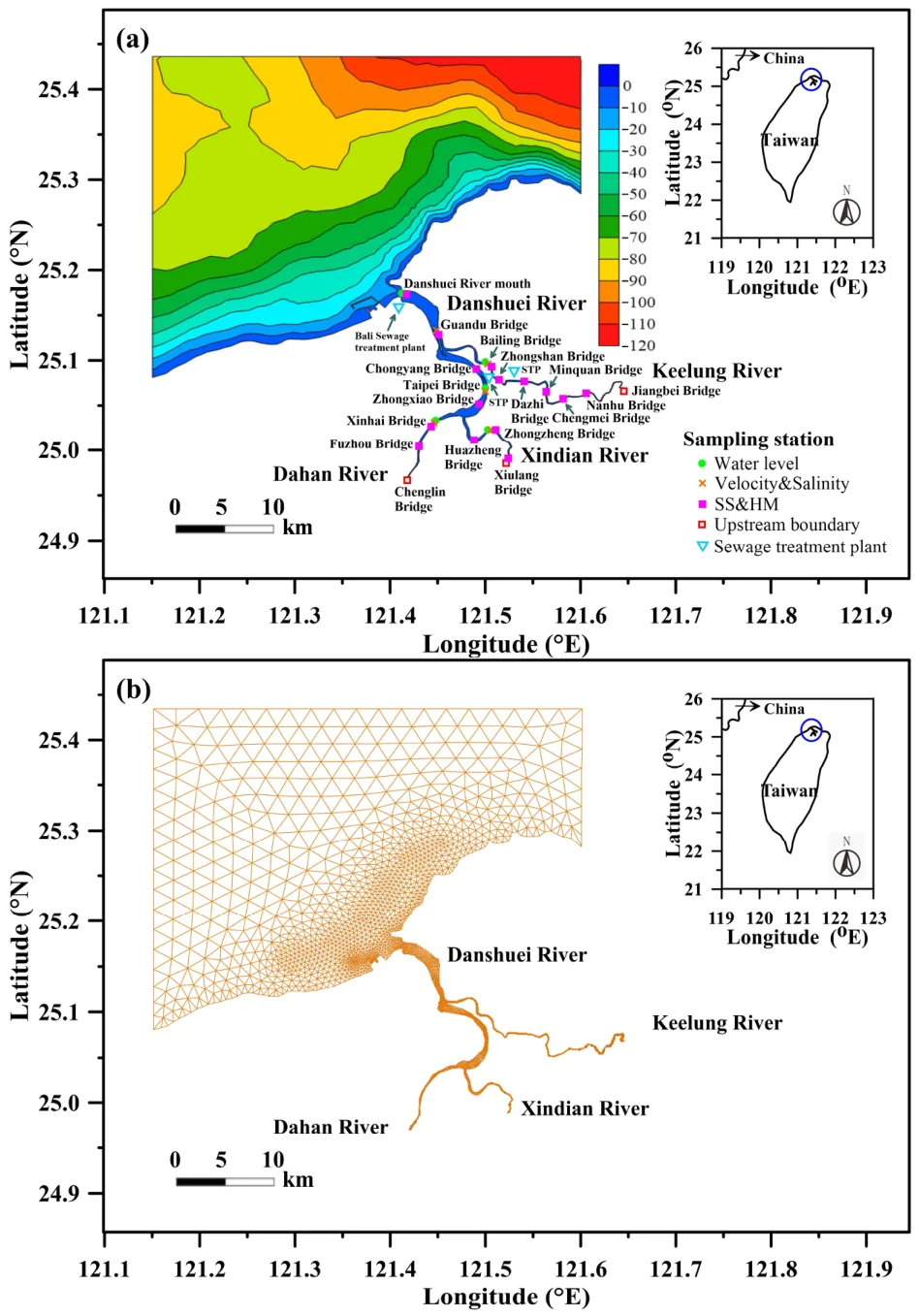
Schematic of the Danshuei River basin showing (**a**) the bathymetry and distribution of monitoring stations and (**b**) the unstructured computational grid used in the SCHISM-HM model.

The Danshuei River flows through densely populated urban areas in northern Taiwan and receives substantial inputs of treated and untreated domestic wastewater. The locations of wastewater treatment plants are shown by blue triangles in Figure 1a. Combined with reduced freshwater discharge during the dry season, these inputs frequently result in environmental problems in the estuarine region, including water quality deterioration, dissolved oxygen depletion, and heavy metal accumulation. According to the 2024 Environmental Water Quality Monitoring Annual Report issued by the Ministry of Environment of Taiwan, the monitored heavy metals showed different levels of compliance with the national water quality standard. Manganese exhibited a compliance rate of only 69.1%, whereas all other monitored heavy metals achieved 100% compliance. These results indicate that manganese represents a comparatively greater heavy metal concern in the Danshuei River basin and therefore was selected as the target contaminant in this study [28].

### 2.2. Data Collection

To develop the three-dimensional hydrodynamic–heavy metal transport model (SCHISM-HM), datasets describing the basin topography, hydrodynamics, and water quality were compiled. River cross-sectional data were obtained from the Tenth River Management Branch of the Water Resources Agency, Ministry of Economic Affairs, while bathymetric data for the adjacent coastal waters were provided by the Ocean Data Bank supported by the National Science and Technology Council, Taiwan. These datasets were integrated to generate the computational mesh, with bed elevations ranging from −119.16 to −0.20 m (Figure 1a).

Hydrodynamic boundary conditions were established using observed river discharges at Chenglin Bridge, Xiulang Bridge, and Jiangbei Bridge. At the offshore open boundary, observed coastal water levels were prescribed, and the principal tidal constituents were obtained through harmonic analysis. Model calibration and validation were performed using observed water levels, current velocities, salinity, suspended sediment concentrations, and total manganese concentrations. Later, for simplicity, manganese concentration is used throughout this paper to refer to the total manganese concentration unless otherwise specified. In total, the observational dataset includes measurements from three discharge stations, five water level stations, five velocity and salinity stations, and fifteen suspended sediment and manganese monitoring stations (Figure 1a). All hydrological and water quality data were obtained from the Water Resources Agency and the Ministry of Environment of Taiwan.

Since 2022, the Ministry of Environment of Taiwan has conducted quarterly sampling and analysis of manganese in the Danshuei River basin. Water samples were collected using a water sampler from the left bank, river center, and right bank at each sampling station and were subsequently mixed in equal volume proportions to obtain a representative composite sample for each sampling location. For water samples collected in the tidal reaches, Mn analysis was performed using ammonium pyrrolidine dithiocarbamate (APDC) chelation–methyl isobutyl ketone (MIBK) extraction followed by atomic absorption spectrophotometry. Samples were not filtered prior to chemical analysis; therefore, dissolved and suspended Mn fractions were not separately determined, and the reported values represent total Mn concentrations. The method detection limit was 0.0059 mg/L.

**Figure 2:**
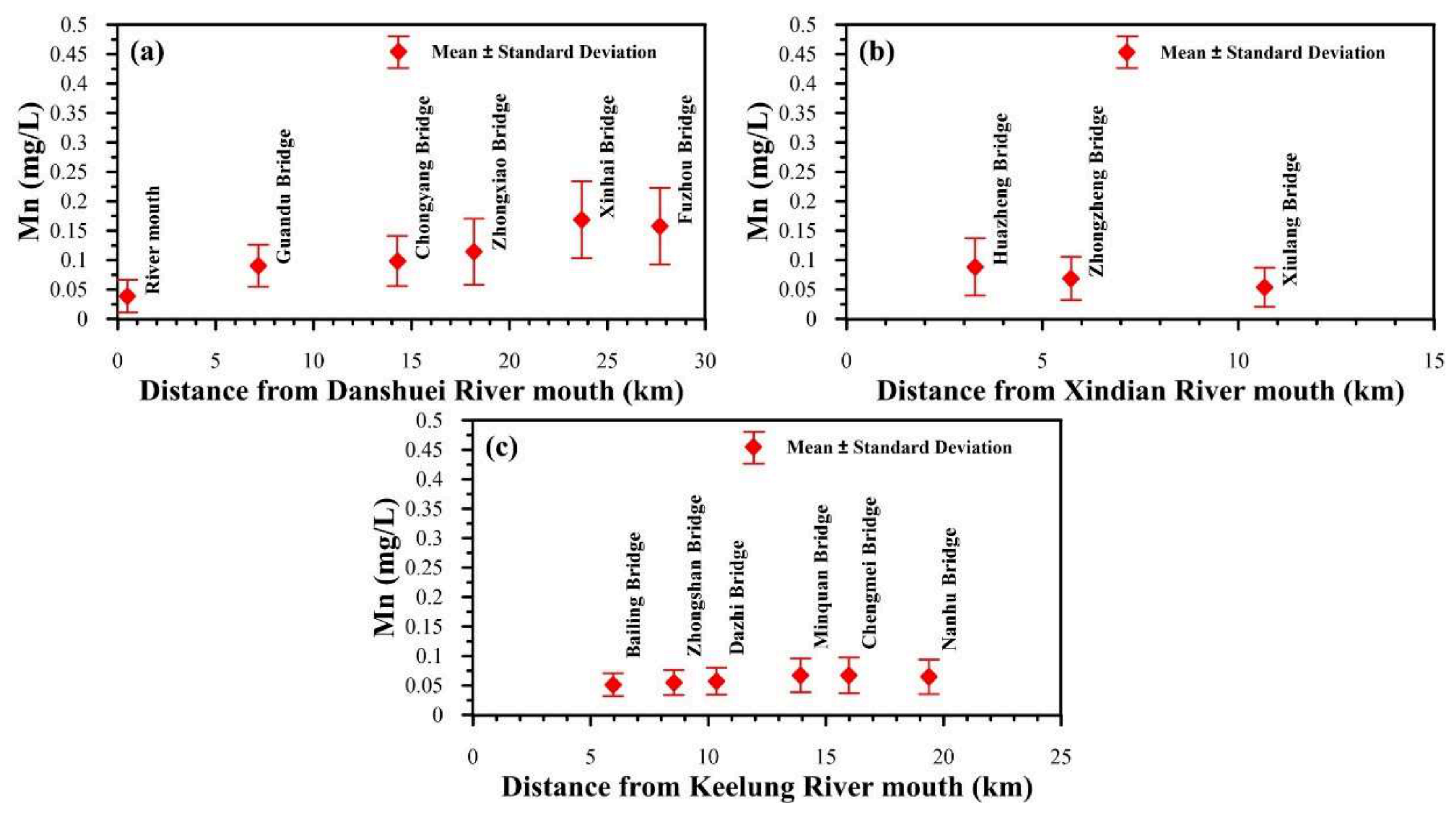
Mean manganese concentrations and corresponding standard deviations during 2014–2023 in (**a**) the Danshuei–Dahan River, (**b**) the Xindian River, and (**c**) the Keelung River.

Figure 2 presents the mean manganese concentrations and corresponding standard deviations at the monitoring stations in the Danshuei River basin during 2014–2023, based on 38 quarterly observations at each station. According to local water quality standards, the manganese concentration limit of 0.05 mg/L, which applies to individual water samples from inland water bodies (i.e., rivers, lakes, and reservoirs), has been established to protect human health. Among all monitoring locations, only the station at the Danshuei River mouth had a mean manganese concentration below the reference value of 0.05 mg/L, with a mean concentration of 0.039 ± 0.028 mg/L. At the remaining stations, mean manganese concentrations ranged from 0.051 to 0.169 mg/L, with standard deviations between 0.019 and 0.065 mg/L. Along the Danshuei–Dahan River, manganese concentrations increased progressively from the estuary toward the upstream reaches, with mean concentrations rising from 0.039 to 0.169 mg/L. Elevated manganese concentrations were also observed in the downstream reach of the Xindian River, where the mean concentration reached 0.088 mg/L, possibly reflecting the influence of manganese-rich inflow from the Dahan River at the confluence. In contrast, manganese concentrations along the Keelung River exhibited relatively limited spatial variation.

## 3. Materials and Methods

### 3.1. Three-Dimensional Hydrodynamic Model

SCHISM (version 5.3.1; Virginia Institute of Marine Science, College of William & Mary, Gloucester Point, VA, USA) employs a semi-implicit hybrid numerical framework that combines finite element and finite volume methods to solve the Navier–Stokes and mass transport equations in a fully coupled manner. In the horizontal plane, an unstructured grid is used to accurately represent complex coastlines and riverine geometries, while a hybrid S–Z vertical coordinate system provides an optimal balance between near-bed resolution and numerical stability in deep-water regions.

For temporal integration, SCHISM adopts a semi-implicit time-stepping scheme coupled with a mode-splitting technique, which relaxes the constraints imposed by the Courant–Friedrichs–Lewy (CFL) condition and improves both computational efficiency and numerical stability. Horizontal momentum is discretized using the finite element method, whereas vertical momentum is solved using the finite volume method. In addition, momentum advection is treated with an Eulerian–Lagrangian approach, which effectively reduces numerical diffusion and enhances solution accuracy, particularly under high-velocity flow conditions.

A detailed description of the governing equations and numerical algorithms is provided by [29]. As an open-source modeling system, SCHISM also allows users to modify the source code and integrate additional process modules, such as water quality and suspended sediment transport, thereby facilitating its application to a wide range of hydrodynamic and environmental studies.

### 3.2. Suspended Sediment Model

Because heavy metals in aquatic environments occur in both dissolved and particulate phases, and the particulate fraction is primarily transported through adsorption onto suspended sediments, a suspended sediment transport module was coupled with the three-dimensional hydrodynamic model SCHISM in this study. Driven by the simulated three-dimensional hydrodynamic fields, the module resolves the advection, diffusion, and vertical mixing of suspended sediments within the water column, thereby characterizing their spatiotemporal distributions. These distributions provide essential input for subsequent heavy metal transport simulations.

In addition to water column processes, the suspended sediment module accounts for sediment exchange at the sediment–water interface. The governing equation for suspended sediment flux at the bed is expressed as follows [30]:

(1) $$w_{s}\mathrm{TSS}\;+\;K_{z}\frac{\partial \mathrm{TSS}}{\partial z}\;=\;D-E\;\mathrm{at}\;z\;=-h$$

where $w_{s}$ represents the settling velocity of suspended sediment (m/s), *TSS* denotes the total suspended sediment concentration (mg/L), *K_z_* expresses the vertical eddy diffusivity coefficient (m^2^/s), *z* indicates the vertical coordinate, positive upwards (m), *D* is the deposition flux (kg/m^2^·s), *E* represents the erosion flux (kg/m^2^·s), and *h* denotes the water depth (m).

The deposition flux (*D*) and erosion flux (*E*) are parameterized as follows:

(2) $$D\;=\;\left\{\begin{array}{ll} w_{s}{\mathrm{TSS}}_{b}\left(1-\frac{\tau_{b}}{\tau_{\mathrm{cd}}}\right) & \mathrm{for}\;\tau_{\mathrm{cd}}>\tau_{b}\\ 0 & \mathrm{for}\;\tau_{\mathrm{cd}}\leq \tau_{b} \end{array}\right.$$

(3) $$E=\left\{\begin{array}{ll} M\left(\frac{\tau_{b}}{\tau_{\mathrm{ce}}}-1\right) & \mathrm{for}\;\;\tau_{b}>\tau_{\mathrm{ce}}\\ \;0 & \mathrm{for}\;\;\tau_{b}\leq \tau_{\mathrm{ce}} \end{array}\right.$$

where ${\mathrm{TSS}}_{b}$ represents the near-bed suspended sediment concentration (mg/L); $\tau_{b}$ denotes the bed shear stress (N/m^2^); $\tau_{\mathrm{cd}}$ and $\tau_{\mathrm{ce}}$ are the critical shear stresses for deposition and erosion (N/m^2^), respectively; and *M* is the erosion rate (kg/m^2^·s).

### 3.3. Heavy Metal Model

To simulate the transport behavior of manganese (Mn) in the Danshuei River estuary, a heavy metal transport module was coupled with the three-dimensional hydrodynamic model SCHISM, resulting in the development of an integrated three-dimensional hydrodynamic–heavy metal transport model (SCHISM-HM). The SCHISM-HM model is designed to represent key processes governing heavy metal dynamics in aquatic systems, including advection, diffusion, deposition, and erosion. The governing equation describing the temporal evolution of heavy metal concentration is expressed as follows:

(4) $$\frac{\partial C}{\partial t}+\frac{\partial \left(\mathrm{uC}\right)}{\partial x}+\frac{\partial \left(\mathrm{vC}\right)}{\partial y}+\frac{\partial \left(\mathrm{wC}\right)}{\partial z}\;=\;\frac{\partial }{\partial x}\left(K_{x}\frac{\partial C}{\partial x}\right)+\frac{\partial }{\partial y}\left(K_{y}\frac{\partial C}{\partial y}\right)+\frac{\partial }{\partial z}\left(K_{z}\frac{\partial C}{\partial z}\right)+\;W\;+\;M_{C}$$

where *C* represents the heavy metal concentration (mg/L); *u*, *v*, and *w* denote the velocity components in the Cartesian coordinate system (*x*, *y*, *z*); $K_{x}$ and $K_{y}$ express the horizontal eddy diffusivity coefficients (m^2^/s); $K_{z}$ indicates the vertical eddy diffusivity coefficient (m^2^/s); *W* represents the external loading from point sources or nonpoint sources (mg/L); and $M_{C}$ is the heavy metal concentration associated with deposition or erosion (mg/L).

Heavy metals readily adsorb onto suspended sediment surfaces; consequently, their concentrations are strongly influenced by bed deposition and erosion processes. The governing equations describing the variation in heavy metal concentration associated with suspended sediment deposition and erosion are given as follows [31]:

(5) $$M_{C}=\frac{E}{{\mathrm{TSS}}_{b}}f_{p}\frac{\partial C}{\partial z}-\frac{D}{\mathrm{TSS}}f_{p}\frac{\partial C}{\partial z}$$

(6) $${\mathrm{TSS}}_{b}=\left(1+\frac{\frac{w_{s}\;\times \;z_{b}}{K_{z}}}{1.25+4.75\;\times {\left(1-\frac{\tau_{b}}{\tau_{\mathrm{cd}}}\right)}^{2.5}}\right)\mathrm{TSS}$$

where $f_{p}$ represents the proportion of heavy metals in particulate form, and $z_{b}$ denotes the thickness of the bottom layer (m).

The partition coefficient (*K_d_*) characterizes the distribution of heavy metals between dissolved and particulate phases. The corresponding governing equations are expressed as follows:

(7) Kd = fpTSSfd

(8) $$f_{p}=\frac{K_{d}\times \mathrm{TSS}}{1+{\;K}_{d}\times \mathrm{TSS}}$$

(9) $$f_{d}=\frac{1}{1+K_{d}\times \mathrm{TSS}}$$

where $K_{d}$ represents the partition coefficient (L/mg), and $f_{d}$ denotes the proportion of heavy metals in dissolved form.

### 3.4. Statistical Analysis

To evaluate the performance of the SCHISM-HM model in simulating hydrodynamics, suspended sediment, and manganese concentrations in the Danshuei River estuary, three statistical metrics were employed to quantify the discrepancies between simulated and observed values. Among the available error measures, mean absolute error (*MAE*), root mean square error (*RMSE*), and skill score (*SS*) were selected as performance indicators. Lower values of *MAE* and *RMSE* indicate smaller deviations between simulations and observations, reflecting improved predictive accuracy of the SCHISM-HM model. In addition, the *SS* provides an overall measure of model performance, with values approaching 1.0 indicating higher simulation accuracy [32]. The corresponding formulations of these statistical metrics are given as follows:

(10) $$\mathrm{MAE}\;=\;\frac{\sum_{i=1}^{N}\left|x_{o,i}-x_{s,i}\right|}{N}$$

(11) $$\mathrm{RMSE}=\sqrt{\frac{\sum_{i=1}^{N}{\left(x_{o,i}-x_{s,i}\right)}^{2}}{N}}$$

(12) $$\mathrm{SS}=\;1-\frac{\sum_{i=1}^{N}{\left|x_{o,i}-x_{s,i}\right|}^{2}}{\sum_{i=1}^{N}{\left(\left|x_{o,i}-{\overset{-}{x}}_{o}\right|+\left|x_{s,i}-{\overset{-}{x}}_{o}\right|\right)}^{2}}$$

where *N* represents the total number of data; $x_{o,i}$ denotes the *i*th observation; $x_{s,i}$ denotes the *i*th simulated value; and ${\overset{-}{x}}_{o}$ is the mean of observations. An *SS* value of 1.0 denotes perfect agreement, values between 0.65 and 1.0 indicate excellent performance, 0.50–0.65 indicate very good performance, 0.20–0.50 indicate good performance, and values below 0.20 indicate poor performance.

### 3.5. Uncertainty Analysis

Monte Carlo simulation (MCS) was employed to quantify the uncertainty of the SCHISM-HM model in simulating manganese transport in the Danshuei River estuary. The key parameters governing heavy metal transport were first identified through data collection and analysis, and their associated uncertainties were characterized by fitting appropriate probability distribution functions. Random samples were subsequently drawn from these distributions to generate parameter sets for ensemble SCHISM-HM simulations, thereby enabling the propagation of parameter uncertainty to model predictions of manganese concentrations.

Among the identified parameters, the partition coefficient (*K_d_*) and the suspended sediment settling velocity (*w_s_*) were found to be the dominant sources of uncertainty in heavy metal transport simulations. Owing to the limited availability of field observations, the parameter ranges were established based on a comprehensive review of the published literature (Table 1 and Table 2), from which the corresponding probability distributions were derived. To ensure consistency with previous studies, the suspended sediment settling velocity was expressed in units of m/day. The compiled datasets indicate that *K_d_* ranges from 0.0001 to 0.4400 L/mg, whereas *w_s_* varies between 2.16 and 11.23 m/day.

To further improve the reliability of uncertainty estimation under limited observational data, Bayesian inference was incorporated into the MCS framework. Bayesian inference updates prior probability distributions using observational evidence to obtain posterior distributions through Bayes’ theorem, thereby reducing parameter uncertainty and improving the consistency between model predictions and observations. This approach has been widely applied in uncertainty analyses of hydrodynamic and water quality models [52,53], and its mathematical formulation is expressed as follows:

(13) $$P\left(\theta \left|Y\right.\right)=\frac{P(Y\left|\theta \right.)P(\theta )}{P(Y)}=\frac{P(Y\left|\theta \right.)P(\theta )}{\int_{\theta }P(Y\left|\theta \right.)P(\theta )d\theta }\propto P(Y\left|\theta \right.)P(\theta )$$

where *P*$\left(\theta \left|Y\right.\right)$ represents the posterior distribution; $P(Y\left|\theta \right.)$ denotes the likelihood function; *P*(*θ*) indicates the prior distribution; and *P*(*Y*) is the normalizing constant.

The formulation and updating procedures of the Bayesian framework are defined as follows:

1. Prior probability distributions: Due to the lack of site-specific observations of the model parameters, the parameter ranges were defined based on the literature values summarized in Table 1 and Table 2. Initial prior probability distributions were established within these ranges, assuming normal distributions for all parameters.
2. Reference values and likelihood function: Because site-specific observations of the model parameters were not available for the Danshuei River, the literature-based parameter values summarized in Table 1 and Table 2 were used as reference values. The likelihood function was used to evaluate the probability of these reference values given specific parameter values. No independent observational error model was specified because the reference values were derived from published parameter values rather than site-specific observations.
3. Posterior probability distributions: Bayesian inference was performed using MATLAB R2024b (MathWorks, Natick, MA, USA) to update the prior distributions based on the likelihood function and obtain the posterior probability distributions. The resulting posterior distributions were fitted using normal distributions.

Parameter ensembles were subsequently sampled from the posterior probability distributions and used as inputs to the SCHISM-HM model to simulate manganese concentrations in the Danshuei River estuary. To determine the appropriate number of Monte Carlo realizations required for uncertainty analysis, the root mean square (*RMS*) value and the estimation confidence interval (*ECI*) were evaluated as convergence criteria [54,55]. The optimal ensemble size was identified when both metrics exhibited stable convergence with increasing simulation runs.

Following the determination of the optimal ensemble size, the simulated manganese concentrations were evaluated using the Nash–Sutcliffe efficiency (*NSE*) and the ratio of the root mean square error to the standard deviation of the observations (*RSR*). Parameter sets that satisfied the “very good” performance criteria proposed by [56], namely *NSE* > 0.75 and *RSR* ≤ 0.50 (Table 3), were retained for subsequent analyses. The retained parameter samples were then fitted with candidate probability distribution functions, and the goodness of fit was assessed using the root mean square error (*RMSE*) and the correlation coefficient (*R*). The best-fitting distributions were subsequently adopted to characterize the posterior uncertainty of the key parameters and to quantify the propagation of parameter uncertainty into simulated manganese concentrations. The corresponding formulations of the evaluation metrics are presented below:

(14) $$\mathrm{RMS}\;=\;\sqrt{\frac{\sum_{i=1}^{N}{{\mathrm{Mn}}_{s,i}}^{2}}{N}}$$

(15) $$\mathrm{ECI}=\frac{\sum_{i=1}^{N}{\mathrm{Mn}}_{s,95\%}-{\mathrm{Mn}}_{s,5\%}}{N}$$

(16) $$\mathrm{NSE}=1-\frac{\sum_{i=1}^{N}{\left({\mathrm{Mn}}_{o,i}-{\mathrm{Mn}}_{s,i}\right)}^{2}}{\sum_{i=1}^{N}{\left({\mathrm{Mn}}_{o,i}-{\overset{-}{\mathrm{Mn}}}_{o}\right)}^{2}}$$

(17) $$\mathrm{RSR}=\frac{\sqrt{\sum_{i=1}^{N}{\left({\mathrm{Mn}}_{o,i}-{\;\mathrm{Mn}}_{s,i}\right)}^{2}}}{\sqrt{\sum_{i=1}^{N}{\left({\mathrm{Mn}}_{o,i}-{\;\overset{-}{\mathrm{Mn}}}_{o}\right)}^{2}}}$$

(18) $$R=\frac{\sum_{i=1}^{N}\left[\left({\mathrm{Mn}}_{o,i}-{\;\overset{-}{\mathrm{Mn}}}_{o}\right)\left({\mathrm{Mn}}_{s,i}-{\overset{-}{\mathrm{Mn}}}_{s}\right)\right]}{\sqrt{\sum_{i=1}^{N}{\left({\mathrm{Mn}}_{o,i}-{\overset{-}{\mathrm{Mn}}}_{o}\right)}^{2}\sum_{i=1}^{N}{\left({\mathrm{Mn}}_{s,i}-{\overset{-}{\mathrm{Mn}}}_{s}\right)}^{2}}}$$

where ${\mathrm{Mn}}_{s,i}$ represents the simulated value for manganese (mg/L); ${\mathrm{Mn}}_{s,95\%}$ denotes the simulated manganese concentration at the 95th percentile (mg/L); ${\mathrm{Mn}}_{s,5\%}$ indicates the simulated manganese concentration at the 5th percentile (mg/L); ${\mathrm{Mn}}_{o,i}$ is the measured value of manganese (mg/L); ${\overset{-}{\mathrm{Mn}}}_{o}$ represents the mean of the measured manganese concentrations (mg/L); and ${\overset{-}{\mathrm{Mn}}}_{s}$ denotes the mean of the simulated manganese concentrations (mg/L).

## 4. Results and Discussion

### 4.1. Model Setup and Validation

The SCHISM-HM model was applied to simulate three-dimensional hydrodynamics and heavy metal transport in the Danshuei River estuary. The computational domain was discretized using an unstructured mesh consisting of 4092 nodes and 6335 triangular elements, with horizontal grid resolutions ranging from approximately 30 m in the estuarine region to 2500 m in the offshore area (Figure 1b). A 10-layer sigma-coordinate system was adopted in the vertical direction. The model was integrated using a time step of 120 s, and the bottom roughness height (z_0_) was specified as 0.5 cm. Boundary conditions included the daily river discharges of the three major upstream tributaries during 2020, measured at Chenglin Bridge, Xiulang Bridge, and Jiangbei Bridge, together with tidal water levels prescribed at the open boundary using nine principal tidal constituents (M_2_, S_2_, K_1_, N_2_, O_1_, K_2_, P_1_, M_4_, and S_a_). The initial and upstream salinity was specified as 0 ppt, whereas the offshore boundary salinity was fixed at 35.0 ppt. Suspended sediment and manganese concentrations at the upstream boundaries were prescribed using monitoring data obtained from the Environmental Water Quality Information database of the Ministry of Environment, Taiwan. A 30-day spin-up period was employed to allow the SCHISM-HM model to reach a quasi-steady state before the analysis period.

**Figure 3:**
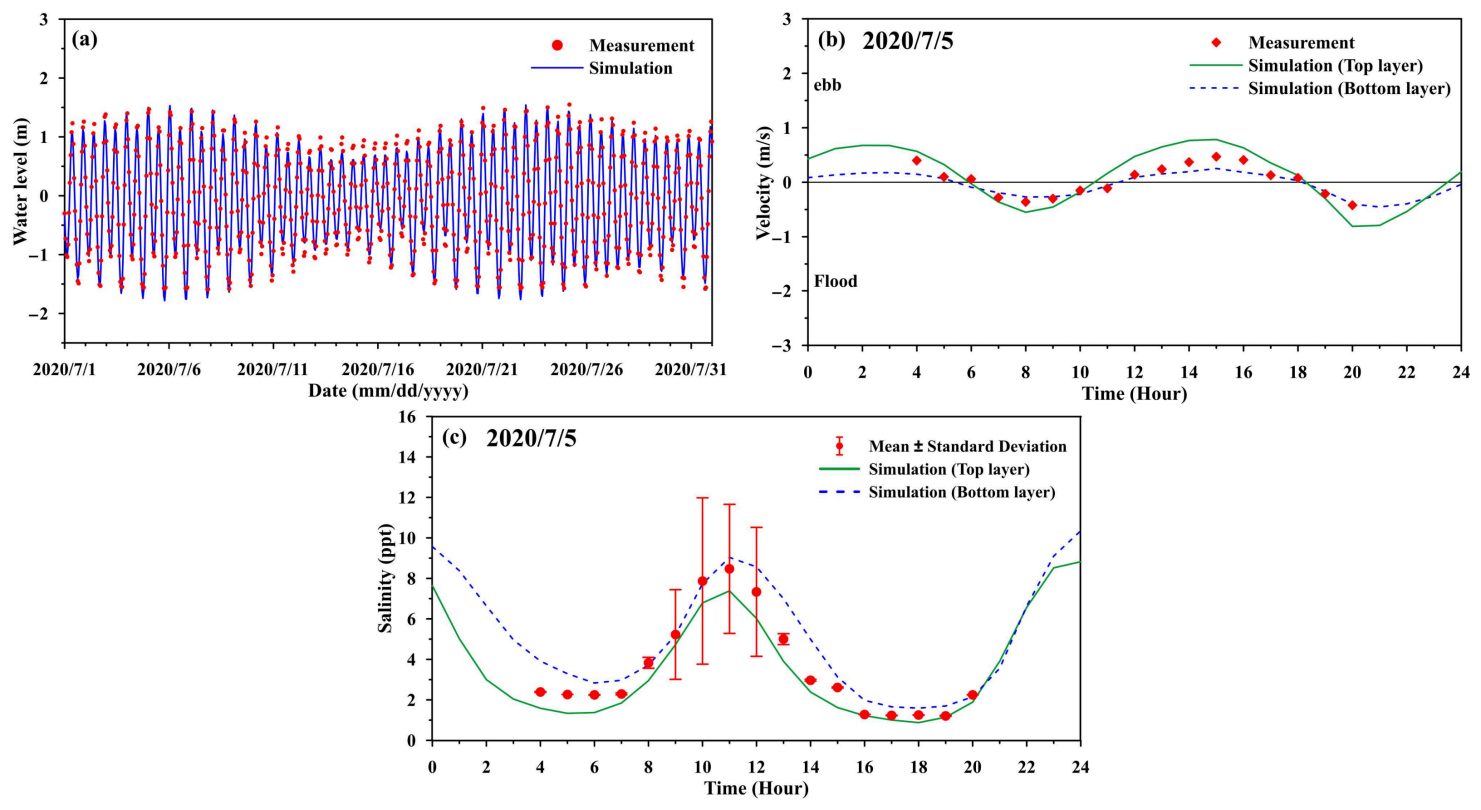
Comparison between observed and simulated (**a**) water level, (**b**) velocity, and (**c**) salinity at the Taipei Bridge station on 5 July 2020.

Model performance was evaluated against field observations collected in January, April, July, and October 2020 for water level, velocity, salinity, suspended sediment, and manganese concentrations. The observations collected in January and April 2020 were used for model calibration, while those collected in July and October 2020 were used for model validation. Hydrodynamic calibration and validation were performed at five monitoring stations. As illustrated in Figure 3, the model accurately reproduced the temporal variations in water level, including both tidal cycles and tidal ranges, with the results at Taipei Bridge on 5 July 2020 presented as a representative example. The model also successfully captured the temporal variations in flow velocity and salinity, including density-driven circulation. Statistical performance metrics (Table 4) further demonstrate the accuracy of the hydrodynamic simulations. For water level, the mean absolute error (*MAE*) ranged from 0.06 to 0.28 m, the root mean square error (*RMSE*) ranged from 0.07 to 0.35 m, and the skill score (*SS*) ranged from 0.952 to 0.998. For velocity, the *MAE* and *RMSE* ranged from 0.04 to 0.15 m/s and 0.05 to 0.17 m/s, respectively, with *SS* values between 0.841 and 0.985. For salinity, the *MAE* ranged from 0.01 to 0.56 ppt, the *RMSE* ranged from 0.01 to 0.84 ppt, and the *SS* ranged from 0.917 to 0.997. These results indicate that the SCHISM-HM model reliably reproduces the hydrodynamic characteristics of the estuarine system.

**Figure 4:**
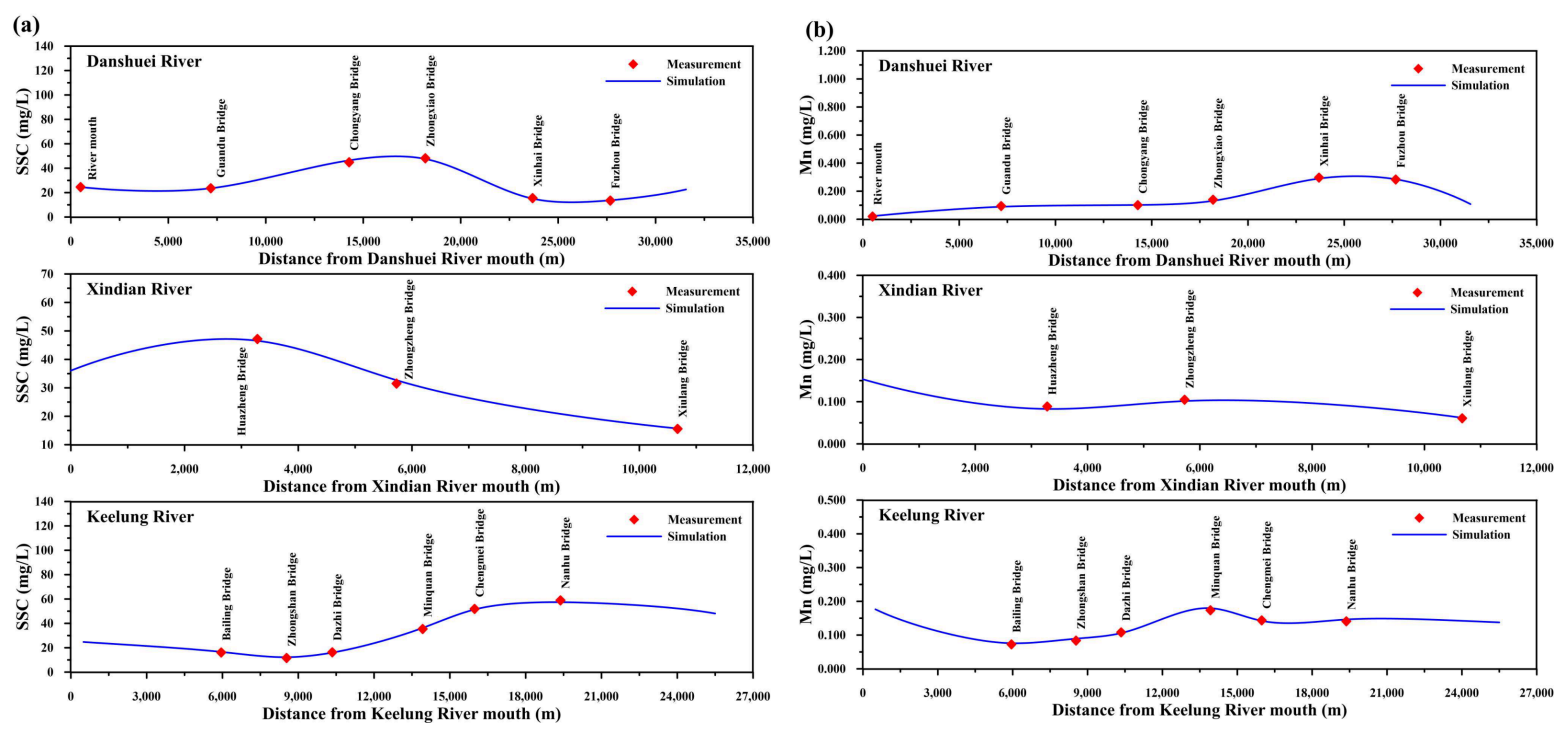
Comparison between observed and simulated (**a**) suspended sediment and (**b**) manganese concentrations at monitoring stations throughout the Danshuei River system on 1 July 2020.

The transport of suspended sediment and manganese was validated using observations from fifteen monitoring stations. As shown in Figure 4, the simulated concentrations agree well with the field measurements, with the results for 1 July 2020 presented as a representative example. Statistical evaluation indicates that, for suspended sediment, the *MAE* ranged from 0.45 to 1.89 mg/L, the *RMSE* ranged from 0.52 to 2.46 mg/L, and the *SS* ranged from 0.893 to 0.999. For manganese, the *MAE* ranged from 0.001 to 0.006 mg/L, the *RMSE* ranged from 0.001 to 0.008 mg/L, and the *SS* ranged from 0.909 to 0.999. Based on the calibration and validation results, the settling velocity (*w_s_*), critical shear stress for deposition ($\tau_{\mathrm{cd}}$), critical shear stress for erosion ($\tau_{ce}$), and erosion rate (*M*) were specified as 7.0 m/day, 1.5 N/m^2^, 2.5 N/m^2^, and 3 × 10^−5^ kg/m^2^·s, respectively. The manganese partition coefficient (*K_d_*) was assigned a value of 0.1 L/mg. Overall, the SCHISM-HM model exhibited strong predictive performance for hydrodynamics, suspended sediment transport, and manganese transport, providing a reliable basis for the subsequent uncertainty analysis.

### 4.2. Hydrodynamic Characteristics and Heavy Metal Distribution

**Figure 5:**
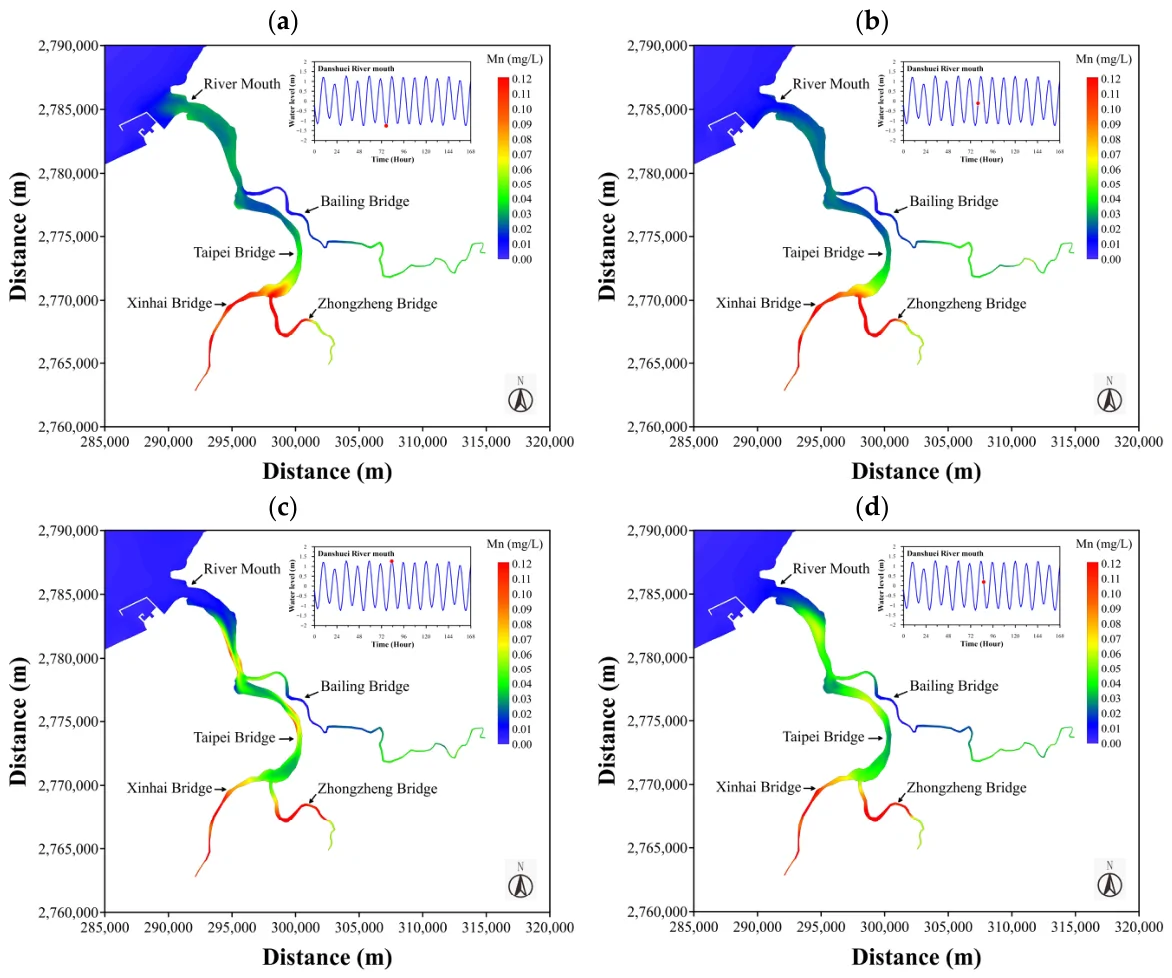
Simulated spatial distribution of manganese concentrations in the Danshuei River estuary during four tidal stages: (**a**) low slack tide, (**b**) maximum flood velocity, (**c**) high slack tide, and (**d**) maximum ebb velocity.

To investigate the influence of tidal dynamics on manganese transport, the simulation results for July 2020, which exhibited the best model validation performance, were selected to analyze the hydrodynamic characteristics and spatiotemporal distribution of manganese in the Danshuei River estuary. Figure 5 presents the simulated spatial distribution of manganese concentrations under different tidal stages. The results indicate that manganese transport is predominantly governed by tidal forcing, resulting in pronounced periodic variations in concentration throughout the estuary. During flood tide, seawater with relatively low manganese concentrations intrudes into the estuary, diluting manganese concentrations in the downstream reaches. Conversely, during ebb tide, river water enriched with manganese is transported from the upstream watershed toward the offshore region, leading to elevated manganese concentrations in the downstream reaches. The simulated tidal variability of manganese concentrations agrees well with the observations reported by [11,20], demonstrating that the SCHISM-HM model can reliably reproduce hydrodynamic transport processes and simulate heavy metal transport under tidal conditions.

A localized zone of elevated manganese concentrations was identified between the estuary and the Taipei Bridge reach. This pattern is primarily attributed to channel morphology and the associated distribution of bed shear stress. As the river channel progressively narrows upstream, current velocities increase during both flood and ebb tides, resulting in enhanced bed shear stress in the shallow nearshore regions. The increased shear stress promotes bed sediment erosion, thereby releasing particulate manganese into the water column [11,12]. Consequently, the temporal variation of manganese concentrations at the Taipei Bridge station differs from that at the other monitoring stations. In particular, the highest manganese concentrations occur during high slack tide rather than low slack tide, whereas peak concentrations at the other stations are primarily associated with upstream river discharge. These findings indicate that local hydrodynamic conditions and sediment–water interactions exert a strong influence on the spatial and temporal distribution of manganese within the estuary. Furthermore, manganese concentrations along the Danshuei River and Dahan River increase progressively from the estuary toward the upstream reaches, consistent with the observed spatial pattern. Overall, the SCHISM-HM model successfully reproduces the coupled hydrodynamic and heavy metal transport processes and captures the combined effects of tidal forcing, riverine transport, and sediment erosion on the distribution of manganese concentrations in the Danshuei River estuary.

### 4.3. Sensitivity Analysis of the Partition Coefficient and Settling Velocity

The spatiotemporal distribution of manganese in the water column is governed not only by hydrodynamic advection and diffusion but also by the settling and resuspension of suspended sediment. Manganese adsorbed onto suspended sediment is transported with sediment particles and subsequently removed from the water column through sediment settling or reintroduced through sediment erosion. Accordingly, the partition coefficient (*K_d_*) and the suspended sediment settling velocity (*w_s_*) were incorporated into the SCHISM-HM model as key parameters to represent the exchange and transport of manganese between the dissolved and particulate phases. To evaluate the sensitivity of model predictions to these parameters, a sensitivity analysis was performed using the July 2020 simulation period. The parameter ranges were selected based on values reported in previous studies of estuarine environments, as summarized in Table 1 and Table 2. Four scenarios were considered for the partition coefficient (*K_d_* = 0.1, 0.2, 0.3, and 0.4 L/mg) and four scenarios for the suspended sediment settling velocity (*w_s_* = 3.0, 5.0, 7.0, and 9.0 m/day). The validated SCHISM-HM model was applied to each scenario, and the resulting variations in manganese concentrations at the monitoring stations were systematically evaluated. For each parameter, four representative values were selected at approximately equal intervals within the plausible range, providing a practical balance between representing the parameter range and maintaining a manageable number of model simulations.

**Figure 6:**
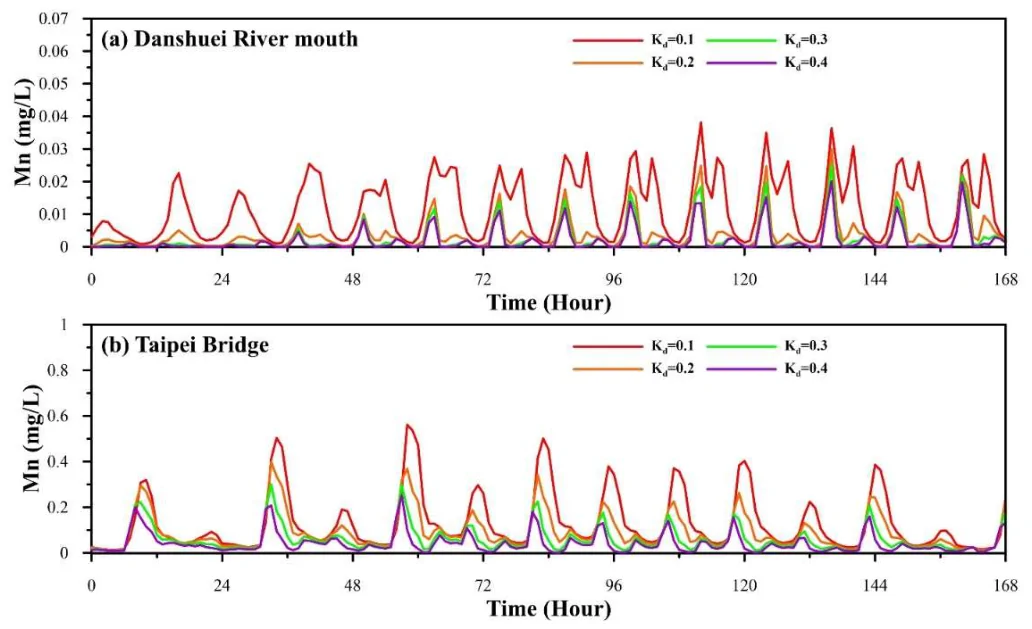

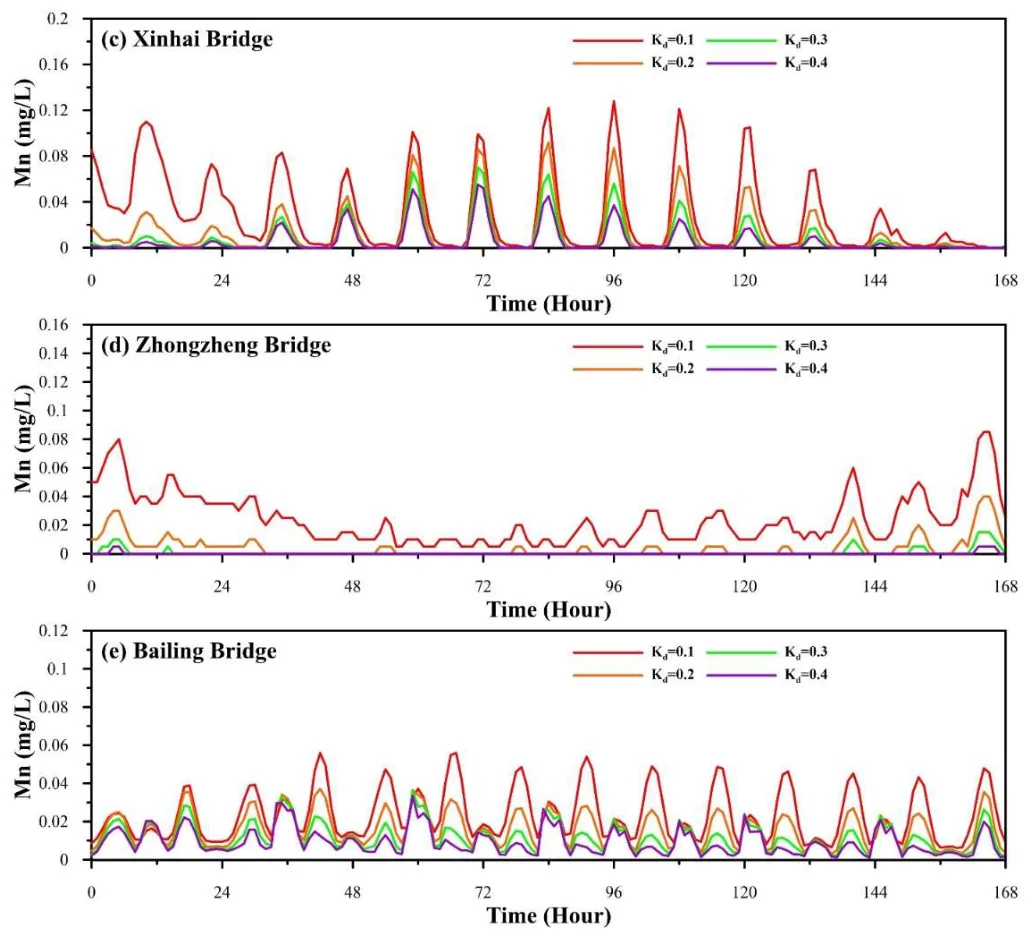
Comparison of simulated hourly manganese concentrations under different partition coefficient (*K_d_*) scenarios.

Figure 6 presents the simulated hourly variations in manganese concentrations under different partition coefficient (*K_d_*) scenarios. The results indicate a strong inverse relationship between *K_d_* and dissolved manganese concentrations in the water column. As *K_d_* increases, a larger fraction of manganese partitions onto suspended sediment, thereby enhancing its removal from the water column through sediment settling and leading to lower dissolved manganese concentrations at all monitoring stations. To quantify the effects of parameter variation, the baseline simulation (*K_d_* = 0.1 L/mg and *w_s_* = 7.0 m/day) was adopted as the reference case. The peak manganese concentration at each monitoring station was identified, and the corresponding percentage change under each *K_d_* scenario was calculated (Table 5). As *K_d_* increased from 0.1 to 0.4 L/mg, the peak manganese concentrations decreased by 32.03–93.75%, demonstrating that the partition coefficient exerts a substantial control on dissolved manganese concentrations in the tidal estuary.

**Figure 7:**
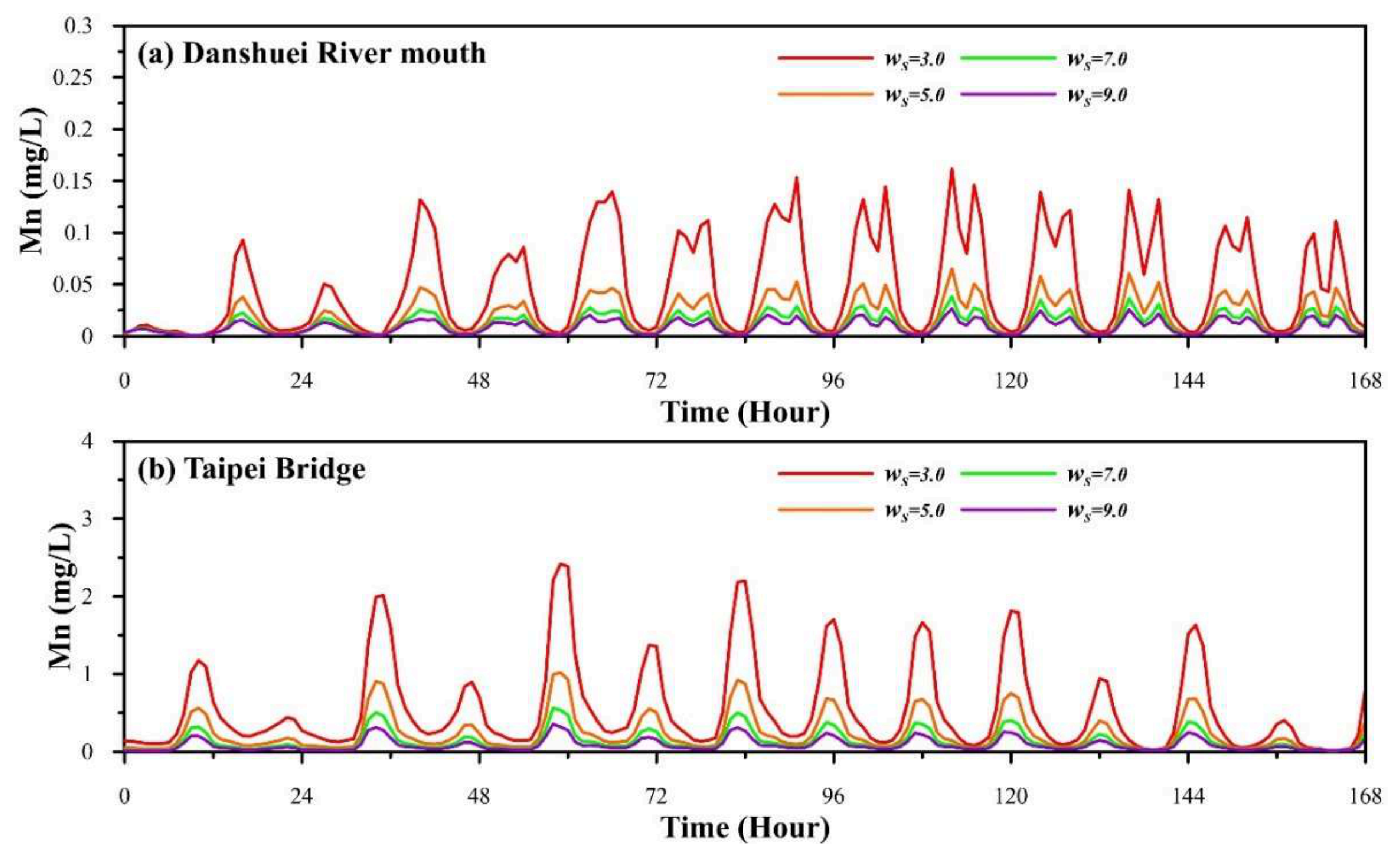

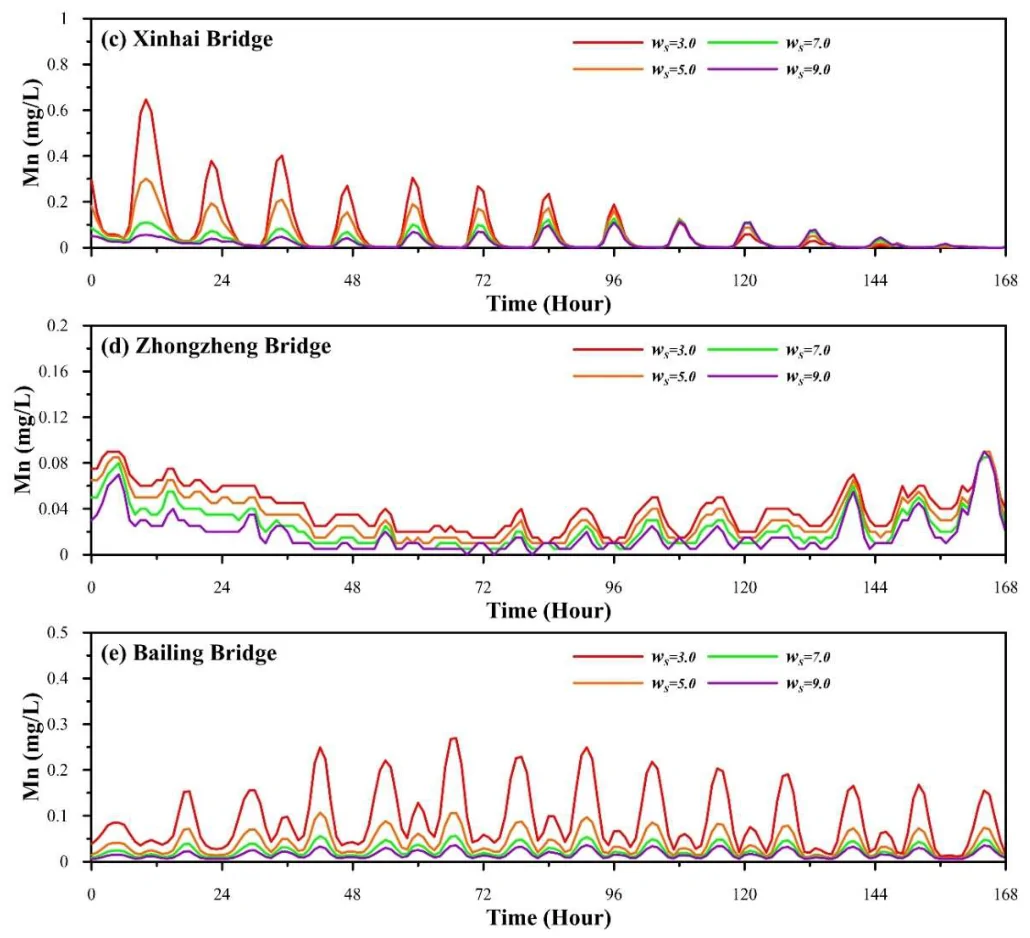
Comparison of simulated hourly manganese concentrations under different settling velocity (*w_s_*) scenarios.

The suspended sediment settling velocity (*w_s_*) also exerted a pronounced influence on manganese transport. Figure 7 compares the simulated hourly manganese concentrations under different settling velocity scenarios. Increasing *w_s_* enhanced the settling of suspended sediment and accelerated the removal of particulate-bound manganese from the water column, resulting in consistently lower manganese concentrations at all monitoring stations. This finding indicates a clear negative relationship between settling velocity and dissolved manganese concentration. The sensitivity of manganese concentrations to settling velocity exhibited distinct spatial variability. Downstream stations, including the Danshuei River mouth, Taipei Bridge, and Bailing Bridge, were considerably more sensitive to changes in *w_s_* than upstream stations. When the settling velocity decreased from the reference value of 7.0 to 3.0 m/day, the reduced sediment settling efficiency substantially increased manganese retention within the water column, resulting in peak concentration increases of 323.34%, 292.58%, and 382.08% at the Danshuei River mouth, Taipei Bridge, and Bailing Bridge stations, respectively. In contrast, the Xinhai Bridge and Zhongzheng Bridge stations exhibited much lower sensitivities, with peak manganese concentrations increasing by only 46.88% and 12.50%, respectively (Table 6). This relatively weak response is likely attributable to the continuous and relatively stable upstream manganese input, which reduces the influence of local variations in sediment settling processes.

Overall, both the partition coefficient (*K_d_*) and the suspended sediment settling velocity (*w_s_*) significantly affect manganese transport in the Danshuei River estuary. However, the model results indicate that manganese concentrations are considerably more sensitive to variations in settling velocity than to changes in the partition coefficient, highlighting the dominant role of suspended sediment dynamics in regulating heavy metal transport in tidal estuaries. These findings are consistent with those of [57], who reported that suspended sediment transport is the primary mechanism controlling the distribution and fate of heavy metals in estuarine environments.

### 4.4. Uncertainty Analysis of Manganese Transport

**Figure 8:**
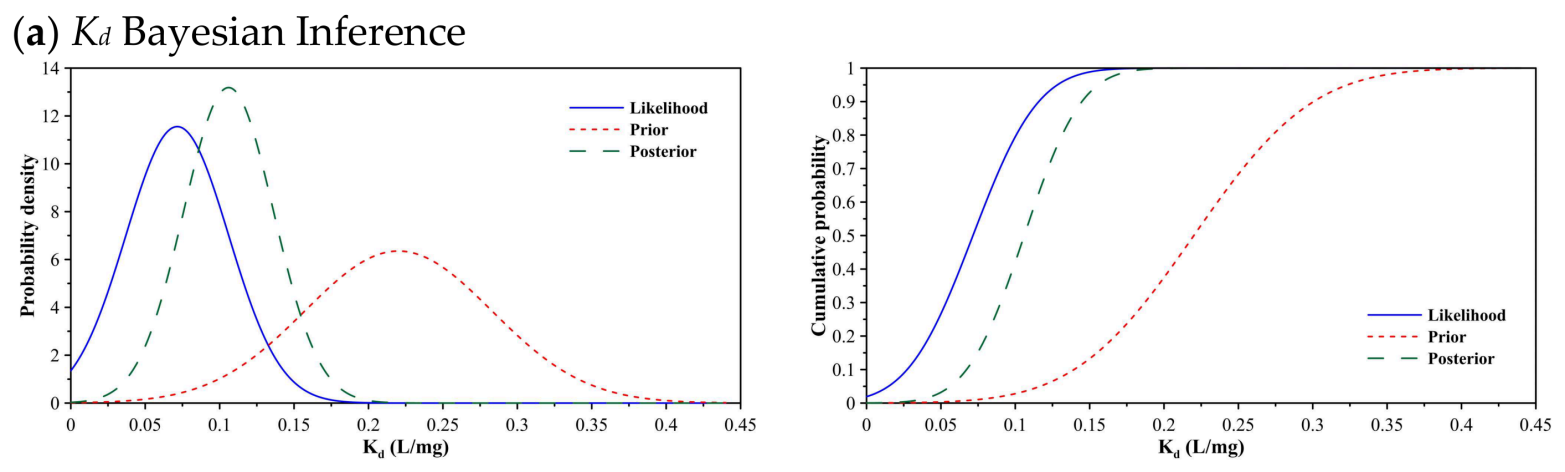

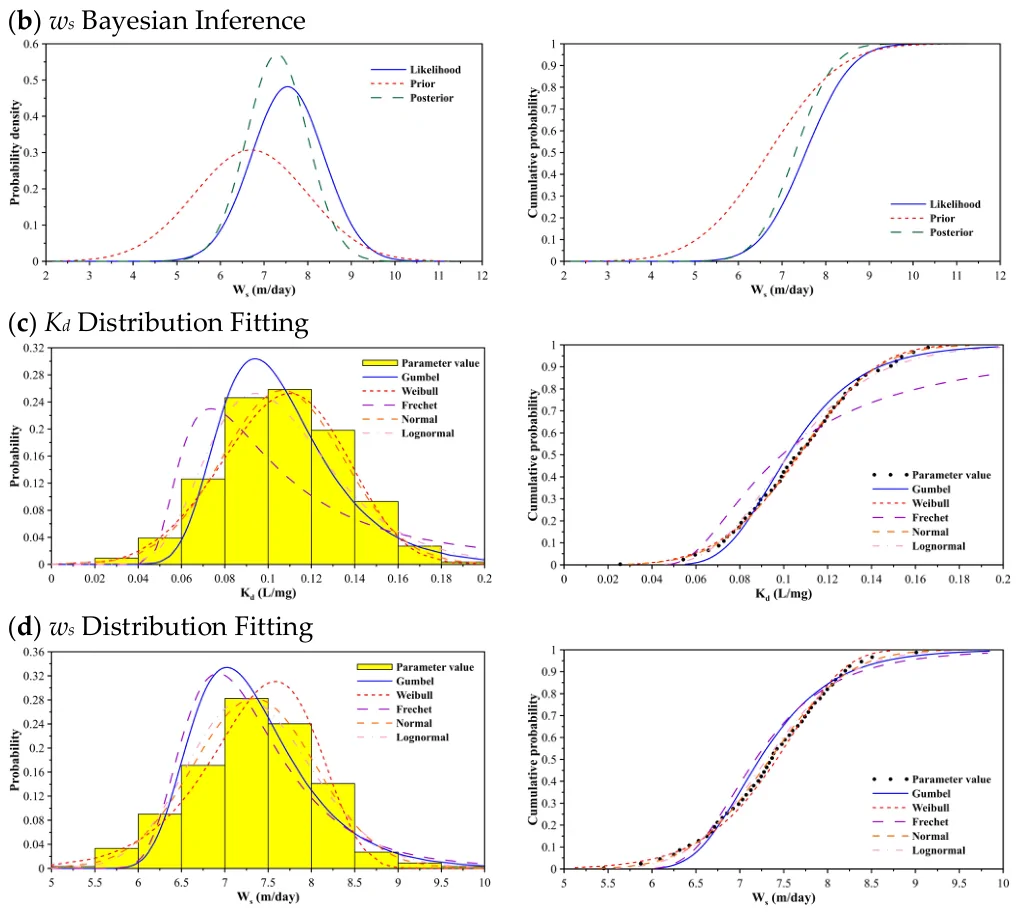
Bayesian updating and probability distribution fitting of the key parameters: (**a**) posterior distribution of the partition coefficient (*K_d_*) obtained through Bayesian inference, (**b**) posterior distribution of the suspended sediment settling velocity (*w_s_*) obtained through Bayesian inference, (**c**) fitted probability density and cumulative distribution functions of the partition coefficient (*K_d_*), and (**d**) fitted probability density and cumulative distribution functions of the suspended sediment settling velocity (*w_s_*).

Tidal estuaries are characterized by complex interactions between upstream river discharge and tidal forcing, resulting in hydrodynamic processes that are considerably more complicated than those in other aquatic environments. Consequently, key parameters controlling heavy metal transport, such as the manganese partition coefficient (*K_d_*) and the settling velocity of suspended sediment (*w_s_*), are difficult to determine directly from field observations, introducing uncertainty into numerical predictions of manganese concentrations. To quantify the influence of these parameter uncertainties on manganese transport in the Danshuei River estuary, the validated July 2020 simulation was selected for uncertainty analysis using a Bayesian–Monte Carlo framework. Based on values reported in the literature, the prior ranges of the partition coefficient (*K_d_*) and suspended sediment settling velocity (*w_s_*) were specified as 0.0001–0.4400 L/mg and 2.16–11.23 m/day, respectively. Bayesian inference was subsequently applied to update the prior distributions and obtain posterior probability distributions that better represent local hydrodynamic and transport conditions, as illustrated in Figure 8a,b. Using these posterior distributions, 2000 random parameter sets were generated through Monte Carlo simulation for ensemble analysis. Ensemble members satisfying the model performance criteria of *NSE* > 0.75 and *RSR* ≤ 0.50 were retained for statistical analysis of the parameter probability distributions. The results, summarized in Table 7, indicate that both *K_d_* and *w_s_* are well described by normal distributions, with *RMSE* values of 0.0015 L/mg and 0.05 m/day, respectively. The corresponding correlation coefficients (*R*) are 0.999 for *K_d_* and 0.997 for *w_s_*, demonstrating excellent agreement between the fitted and posterior probability distributions (Figure 8c,d).

**Figure 9:**
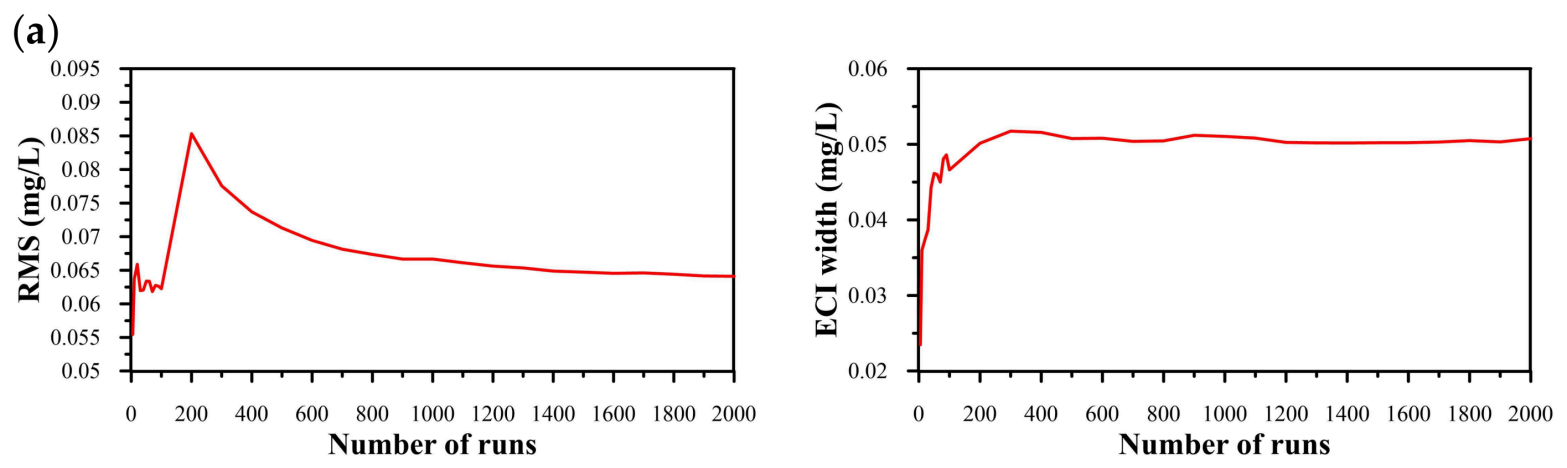

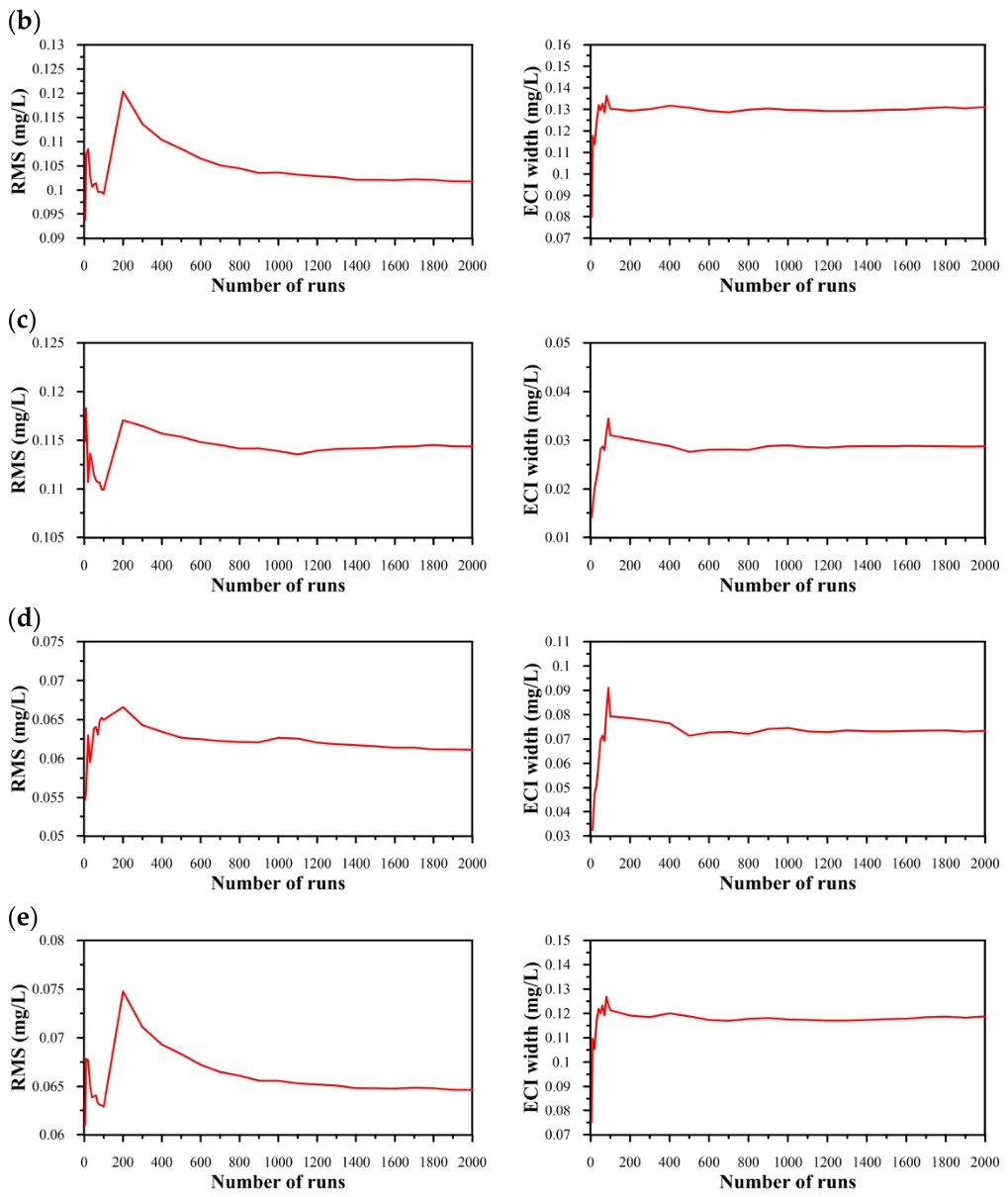
Convergence of the root mean square (*RMS*) and 90% estimation confidence interval (*ECI*) width of simulated manganese concentrations with increasing numbers of Monte Carlo simulations at (**a**) the Danshuei River mouth, (**b**) Taipei Bridge, (**c**) Xinhai Bridge, (**d**) Zhongzheng Bridge, and (**e**) Bailing Bridge.

**Figure 10:**
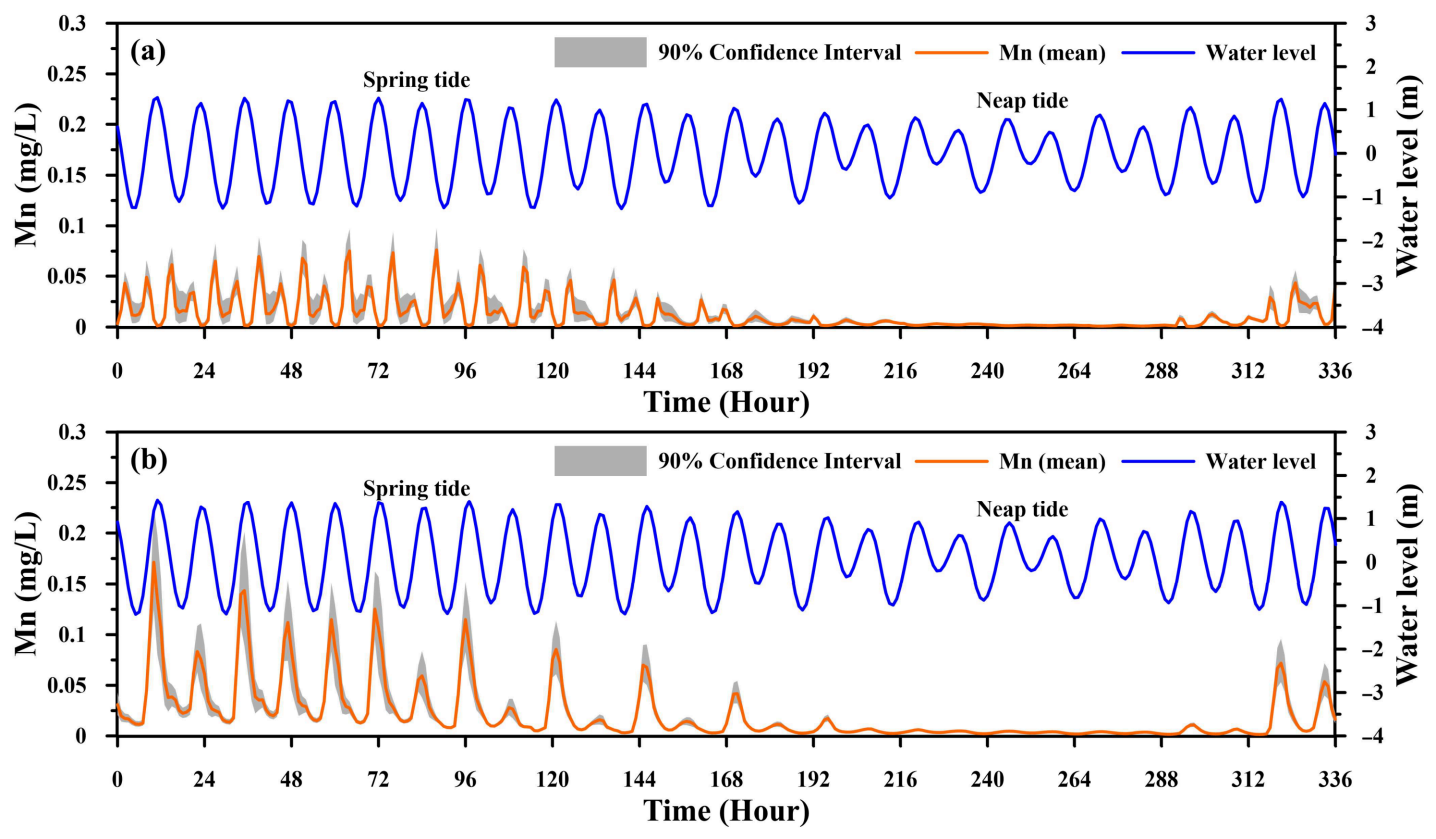

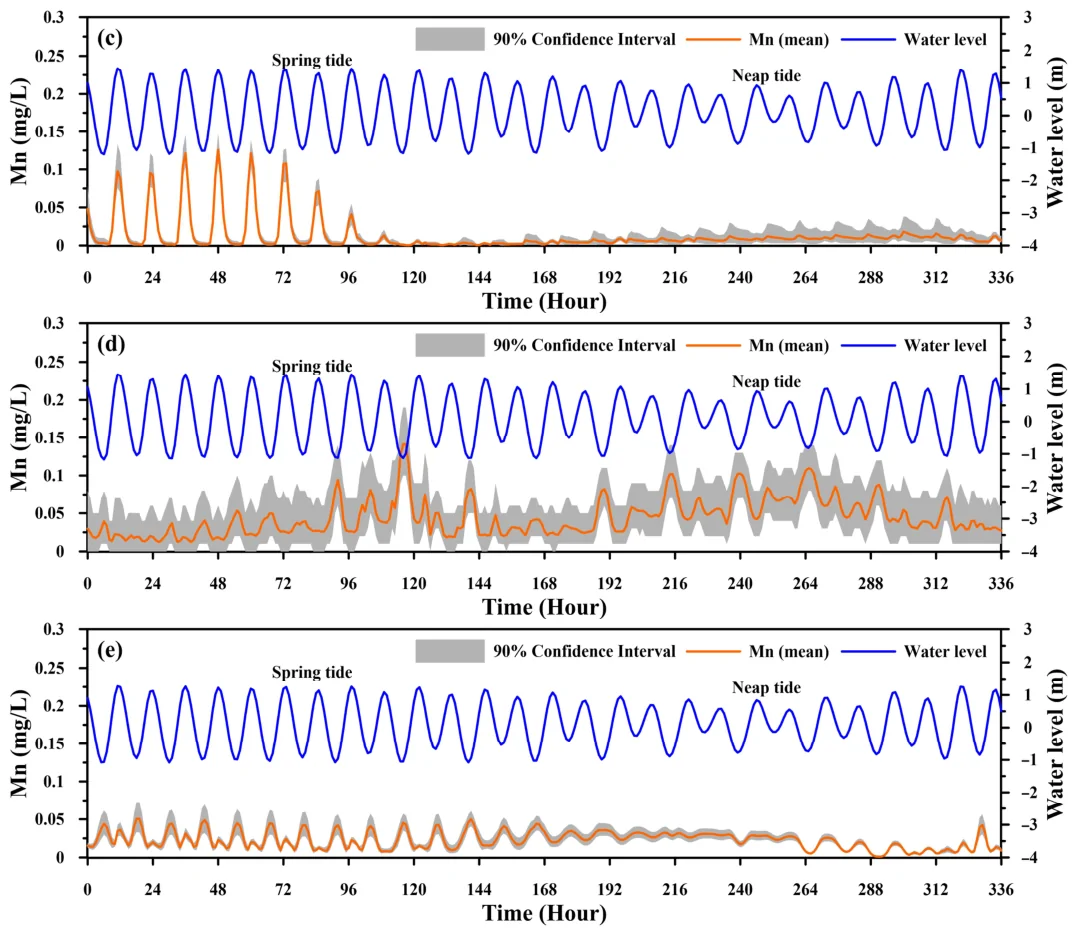
Temporal variations in simulated manganese concentrations and their associated 90% estimation confidence intervals (*ECIs*) at (**a**) the Danshuei River mouth, (**b**) Taipei Bridge, (**c**) Xinhai Bridge, (**d**) Zhongzheng Bridge, and (**e**) Bailing Bridge.

Figure 9 presents the root mean square (*RMS*) values and the 90% estimation confidence intervals (*ECIs*) obtained at each monitoring station for different ensemble sizes. Both the *RMS* values and *ECI* widths converge as the number of simulations approaches approximately 800, indicating that, for the adopted SCHISM-HM model configuration and the present Danshuei River application, an ensemble size of 800 simulations is sufficient to obtain stable uncertainty estimates for manganese concentrations. Figure 10 further demonstrates that manganese concentrations exhibit pronounced periodic fluctuations associated with tidal cycles, consistent with the heavy metal transport characteristics reported by [57]. Because manganese transport is strongly coupled with suspended sediment dynamics, even relatively small variations in *K_d_* and *w_s_* can produce appreciable differences in simulated manganese concentrations. Under tidal forcing, the uncertainty bands are primarily concentrated around the concentration peaks, reflecting the periods during which parameter uncertainty exerts the greatest influence on transport processes.

Among the monitoring stations, the Taipei Bridge station exhibits the largest parameter-induced uncertainty, with a maximum 90% *ECI* width of 0.118 mg/L (Figure 10). This elevated uncertainty is primarily attributed to the local contraction of the river channel, where increased flow velocities generate high bed shear stresses that enhance sediment resuspension and release manganese adsorbed onto bed sediments into the water column [58]. In contrast, the upstream Zhongzheng Bridge station is less influenced by tidal forcing and exhibits a smaller uncertainty concentration, with a maximum 90% *ECI* width of 0.100 mg/L. The remaining monitoring stations, including the Danshuei River mouth (0.039 mg/L), Xinhai Bridge (0.057 mg/L), and Bailing Bridge (0.040 mg/L), display comparatively narrower confidence intervals, indicating that simulated manganese concentrations at these locations are less sensitive to uncertainties in the partition coefficient and settling velocity.

**Figure 11:**
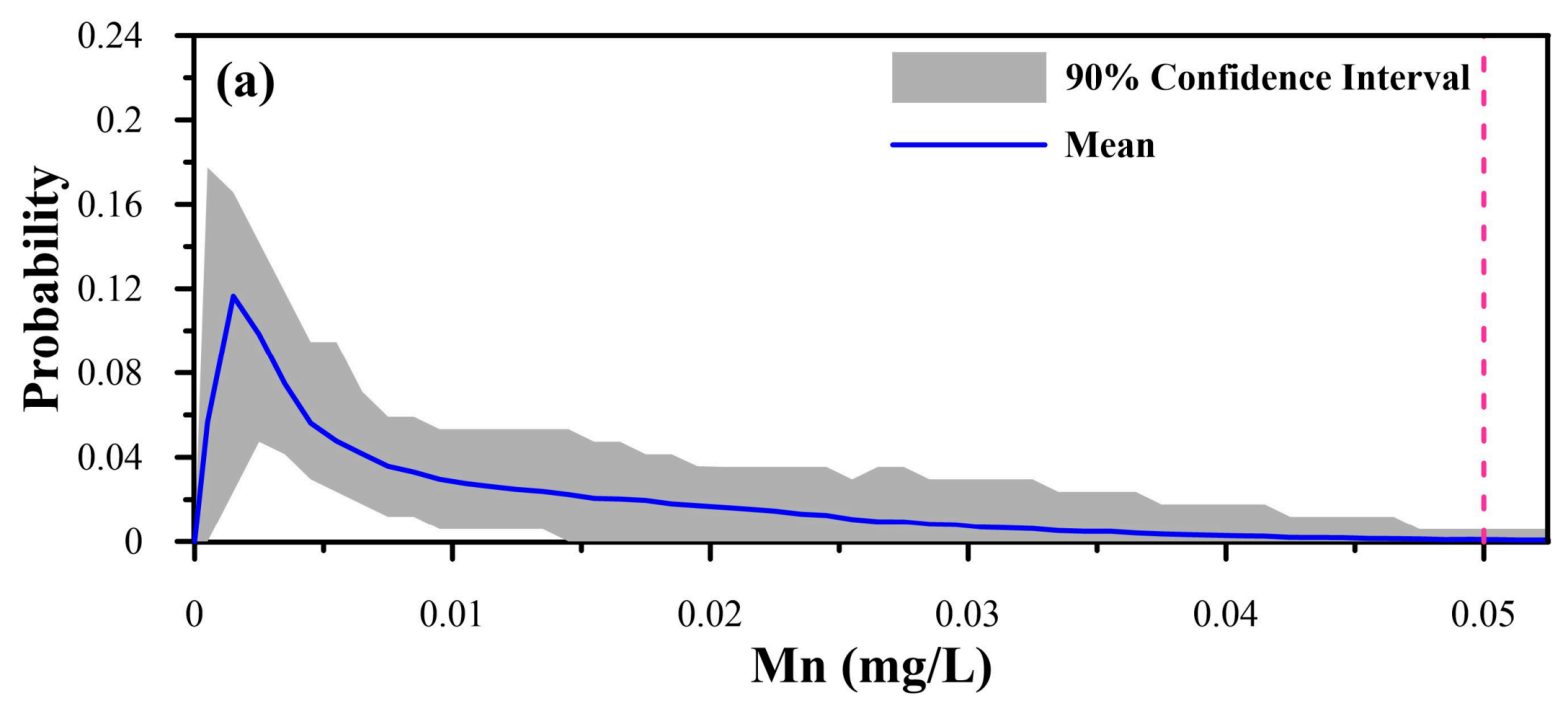

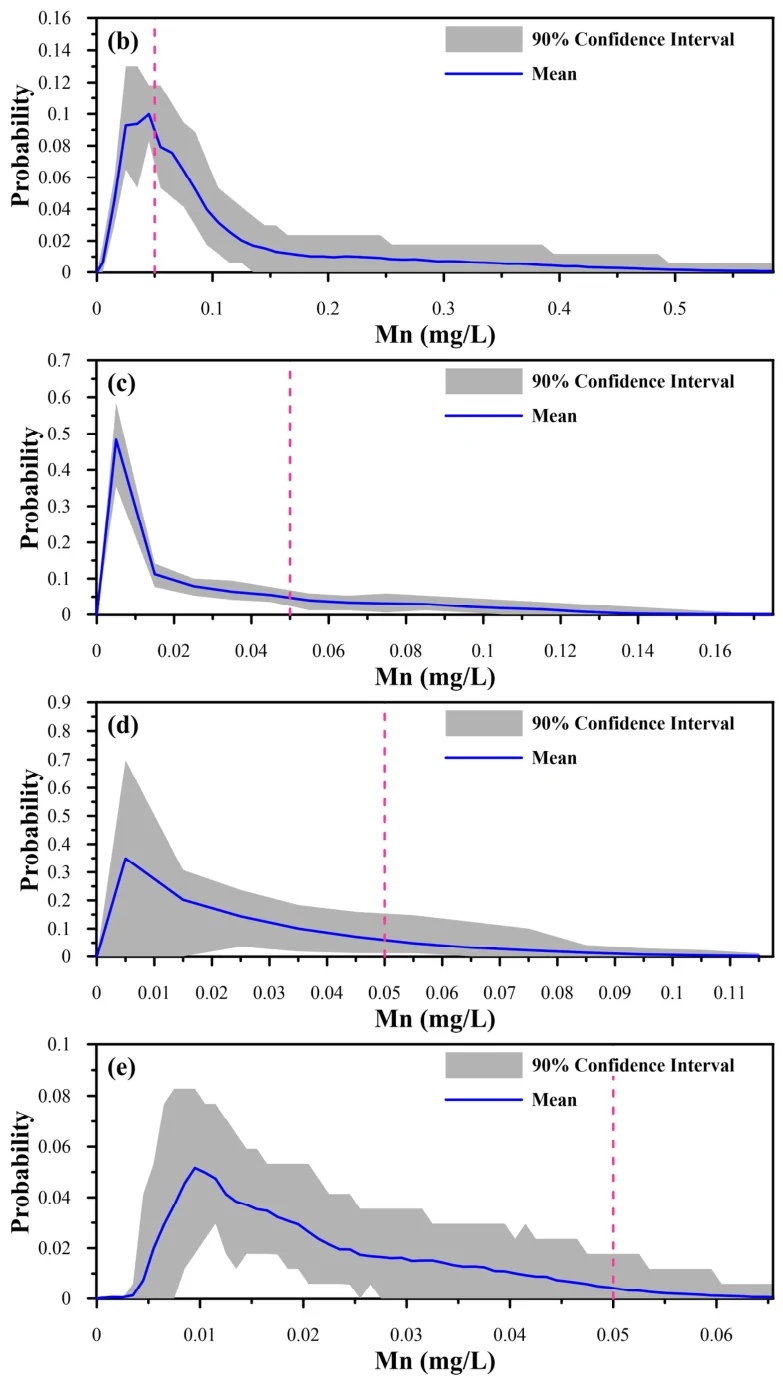
Probability distributions of simulated manganese concentrations and their associated uncertainty at (**a**) the Danshuei River mouth, (**b**) Taipei Bridge, (**c**) Xinhai Bridge, (**d**) Zhongzheng Bridge, and (**e**) Bailing Bridge.

Figure 11 illustrates the uncertainty associated with the occurrence probabilities of the mean manganese concentrations at each monitoring station. The confidence interval widths generally increase with occurrence probability, indicating that the greatest uncertainty is concentrated near the peak of the probability distributions. Nevertheless, uncertainties in *K_d_* and *w_s_* exert only a limited influence on the overall shape of the probability distributions, suggesting that the spatial and temporal patterns of manganese occurrence remain robust despite parameter variability. For reference purposes (rather than to assess regulatory compliance), comparison with the current Taiwanese water quality standard for manganese (0.05 mg/L, indicated by the red dashed line in Figure 11) indicates that the probabilities of the mean manganese concentration exceeding this threshold are 0.7%, 66.3%, 20.6%, 13.3%, and 3.2% at the Danshuei River mouth, Taipei Bridge, Xinhai Bridge, Zhongzheng Bridge, and Bailing Bridge stations, respectively. The highest exceedance probability occurs at Taipei Bridge, indicating that this station has the greatest likelihood of exceeding the water quality standard.

Overall, the integration of Bayesian inference with Monte Carlo simulation provides a robust probabilistic framework for quantifying uncertainty in manganese transport within the Danshuei River estuary. The proposed approach not only estimates confidence intervals for simulated manganese concentrations but also characterizes the probability of threshold exceedance, thereby providing scientifically rigorous information to support watershed water quality management, environmental risk assessment, and evidence-based decision-making.

### 4.5. Limitations and Future Research

Despite the satisfactory performance of the SCHISM-HM model in reproducing the spatiotemporal distribution of manganese in the Danshuei River estuary, several limitations remain regarding parameter estimation, observational data availability, and the broader applicability of the proposed uncertainty assessment framework. First, the prior distributions of the key parameters, namely the partition coefficient (*K_d_*) and suspended sediment settling velocity (*w_s_*), were primarily derived from values reported in the literature rather than from site-specific field measurements conducted in the Danshuei River. Because salinity gradients and suspended sediment characteristics vary considerably among tidal estuaries [20], literature-based parameter distributions may not fully capture the local transport characteristics of manganese within the study area. In addition, the constant partition coefficient (*K_d_*) adopted in the SCHISM-HM model does not account for the potential effects of physicochemical conditions, such as pH, oxidation–reduction potential (ORP), and temperature, on Mn release and partitioning between sediment and water. Previous studies have indicated that these physicochemical factors may influence the mobility and release of elements from sediments [59]. Therefore, incorporating the dependence of *K_d_* on relevant physicochemical conditions may improve the representation of Mn–sediment interactions and reduce uncertainty in model predictions. Future studies should consider developing a dynamic *K_d_* formulation that accounts for variations in these physicochemical conditions to improve the predictive capability of the SCHISM-HM model.

The uncertainty analysis presented in this study focused exclusively on random sampling of the partition coefficient (*K_d_*) and suspended sediment settling velocity (*w_s_*). Other sediment-related parameters in the SCHISM-HM model, including the erosion rate (*M*), critical shear stress for deposition ($\tau_{\mathrm{cd}}$), and critical shear stress for erosion ($\tau_{\mathrm{ce}}$), were calibrated during model calibration and subsequently treated as deterministic values. However, the results in Section 4.4 indicate that manganese concentrations at the Taipei Bridge station are strongly influenced by bed sediment erosion processes. Consequently, excluding these sediment-related parameters from the uncertainty analysis may lead to an underestimation of the overall predictive uncertainty associated with sediment transport processes. Furthermore, the reliability of the uncertainty assessment is constrained by the spatial coverage and temporal frequency of field observations. The current quarterly monitoring program is insufficient to capture short-term fluctuations in manganese concentrations driven by tidal dynamics and episodic hydrological events. In addition, the relative contributions of bed sediment erosion, riverine inputs, tidal transport, and dilution to the elevated Mn concentrations at the Taipei Bridge station were not separately quantified in this study. Additional scenario simulations with erosion suppressed or varied, together with source and mass budget analyses, are needed to further verify the proposed mechanism and will be considered in future research.

Although the convergence analysis demonstrated that 800 Monte Carlo simulations were sufficient to achieve stable uncertainty estimates, this conclusion was obtained for a single heavy metal species during a specific simulation period. The general applicability of this finding therefore requires further validation. Field observations indicate that manganese had a lower compliance rate with the water quality standard than the other monitored heavy metals in the Danshuei River estuarine system. Nevertheless, whether the uncertainty characteristics identified in this study are transferable to other heavy metals, such as copper, zinc, and nickel, remains unclear. Likewise, the Taipei Bridge station exhibited the largest 90% confidence interval width (0.118 mg/L) and the highest probability of exceeding the water quality standard. Whether this behavior is representative of other tidal estuaries with similar convergent channel geometries requires further investigation through comparative studies encompassing a wider range of estuarine geomorphic and hydrodynamic settings. Although the SCHISM-HM framework and the uncertainty assessment approach can be applied to other tidal estuaries, several model inputs and parameters are inherently site-specific. Bathymetry, river discharge, tidal forcing, salinity conditions, sediment properties, and the parameter values governing manganese partitioning and settling should be redefined based on local observations and environmental conditions. Differences in tidal regimes, freshwater inflows, sediment characteristics, and channel morphology among estuaries may also affect manganese transport processes and model responses. Therefore, application of the proposed framework to other estuarine systems requires site-specific parameterization, calibration, and validation using adequate hydrodynamic, sediment, and manganese observations.

Future research should address these limitations by extending the uncertainty assessment framework in several aspects. In addition to the partition coefficient (*K_d_*) and suspended sediment settling velocity (*w_s_*), sediment-related parameters, including the erosion rate (*M*), critical shear stress for deposition ($\tau_{\mathrm{cd}}$), and critical shear stress for erosion ($\tau_{\mathrm{ce}}$), should be incorporated into the stochastic sampling framework to provide a more comprehensive evaluation of uncertainty propagation associated with sediment transport and resuspension processes. Furthermore, the proposed framework should be applied to additional heavy metals, such as copper, zinc, and nickel, and validated across multiple tidal estuaries with contrasting geomorphic and hydrodynamic characteristics. Such investigations would improve the robustness, transferability, and generalizability of the proposed methodology, thereby enhancing the application of numerical models for uncertainty quantification in heavy metal transport, water quality risk assessment, environmental monitoring, and estuarine pollution management.

## 5. Conclusions

This study developed a three-dimensional hydrodynamic–heavy metal transport model (SCHISM-HM) for tidal estuaries and applied it to the Danshuei River estuarine system in Taiwan to investigate manganese transport mechanisms, parameter sensitivity, and predictive uncertainty. The major conclusions are summarized as follows:

1. The SCHISM-HM model satisfactorily reproduced the spatiotemporal variations in water level, flow velocity, salinity, suspended sediment concentration, and manganese concentration in the Danshuei River system, with generally good agreement between simulated and observed data. The results demonstrate that the model can reasonably represent the coupled hydrodynamic, suspended sediment, and manganese transport processes in the study area.
2. The model results indicate that sediment-related processes play an important role in manganese transport. In particular, the elevated manganese concentrations simulated near Taipei Bridge were associated with enhanced sediment erosion under locally increased flow velocity and bed shear stress. However, the relative contributions of bed sediment erosion, riverine inputs, tidal transport, and dilution were not separately quantified, and further scenario simulations and source or mass budget analyses are needed to verify the proposed mechanism.
3. Sensitivity analysis showed that both the partition coefficient (*K_d_*) and suspended sediment settling velocity (*w_s_*) were negatively associated with manganese concentrations in the water column. The influence of *w_s_* was more pronounced than that of *K_d_*, indicating that suspended sediment dynamics have an important influence on manganese transport in the Danshuei River estuary.
4. The uncertainty analysis showed that Monte Carlo simulation combined with Bayesian inference can quantify the uncertainty and occurrence probability of simulated manganese concentrations. For the present Danshuei River application and the adopted SCHISM-HM model configuration, approximately 800 simulations were sufficient to obtain stable uncertainty estimates. The uncertainty was most pronounced during periods of peak manganese concentration, with substantial spatial variability among monitoring stations. Taipei Bridge exhibited the widest confidence interval and the highest probability of the mean manganese concentration exceeding the 0.05 mg/L water quality standard.

Overall, the results highlight the importance of sediment dynamics and key sediment-related parameters in manganese transport and provide a quantitative assessment of predictive uncertainty for the study area.

## Figures and Tables

**Table 1:** Summary of partition coefficients (*K_d_*).

Type of Aquatic Environment	*K_d_* (L/mg)	Reference
Estuary/Coastal	0.0030–0.0070	[33]
Estuary/Coastal	0.0060	[34]
Estuary/Coastal	0.0269–0.3610	[35]
Estuary/Coastal	0.0001–0.0513	[36]
Estuary/Coastal	0.0010–0.2000	[37]
Estuary/Coastal	0.0075	[38]
Estuary/Coastal	0.0010–0.0100	[39]
Coastal	0.0024	[40]
Estuary/Coastal	0.0050	[16]
Estuary/Coastal	0.0001–0.4400	[20]

**Table 2:** Summary of suspended sediment settling velocity (*w_s_*).

Type of Aquatic Environment	*w_s_* (m/day)	Reference
Estuary/Coastal	2.16	[41]
Estuary/Coastal	5.18	[42]
Estuary/Coastal	8.64	[43]
Coastal	8.64	[44]
Estuary/Coastal	8.64	[45]
Estuary/Coastal	8.64	[46]
Estuary/Coastal	5.00	[47]
Estuary/Coastal	8.64	[48]
Estuary/Coastal	8.64	[49]
Estuary/Coastal	11.23	[50]
Estuary/Coastal	8.64	[51]

**Table 3:** Performance evaluation criteria for *NSE* and *RSR*.

Performance Rating	*NSE*	*RSR*
Very good	0.75 < *NSE* ≤ 1.00	0.00 ≤ *RSR* ≤ 0.50
Good	0.65 < *NSE* ≤ 0.75	0.50 < *RSR* ≤ 0.60
Satisfactory	0.50 < *NSE* ≤ 0.65	0.60 < *RSR* ≤ 0.70
Unsatisfactory	*NSE* ≤ 0.50	*RSR* > 0.70

Adapted from [56].

**Table 4:** Statistical performance metrics for model calibration and validation.

Variable	*MAE*	*RMSE*	*SS*
Water level	0.06 m–0.28 m	0.07 m–0.35 m	0.952–0.998 (Excellent)
Velocity	0.04 m/s–0.15 m/s	0.05 m/s–0.17 m/s	0.841–0.985 (Excellent)
Salinity	0.01 ppt–0.56 ppt	0.01 ppt–0.84 ppt	0.917–0.997 (Excellent)
Suspended sediment	0.45 mg/L–1.89 mg/L	0.52 mg/L–2.46 mg/L	0.893–0.999 (Excellent)
Manganese	0.001 mg/L–0.006 mg/L	0.001 mg/L–0.008 mg/L	0.909–0.999 (Excellent)

**Table 5:** Percentage changes in simulated peak manganese concentrations under different partition coefficient (*K_d_*) scenarios.

Station	Partition Coefficient *K_d_* (L/mg)			
	0.1	0.2	0.3	0.4
Danshuei River mouth	0.00%	−34.82%	−51.91%	−64.81%
Taipei Bridge	0.00%	−34.39%	−65.57%	−82.26%
Xinhai Bridge	0.00%	−32.03%	−56.25%	−71.09%
Zhongzheng Bridge	0.00%	−62.50%	−87.50%	−93.75%
Bailing Bridge	0.00%	−46.91%	−75.10%	−88.44%

**Table 6:** Percentage changes in simulated peak manganese concentrations under different suspended sediment settling velocity (*w_s_*) scenarios.

Station	Settling Velocity *w_s_* (m/day)			
	3.0	5.0	7.0	9.0
Danshuei River mouth	323.34%	71.05%	0.00%	−30.62%
Taipei Bridge	292.58%	76.92%	0.00%	−36.40%
Xinhai Bridge	46.88%	25.78%	0.00%	−14.06%
Zhongzheng Bridge	12.50%	6.25%	0.00%	−12.50%
Bailing Bridge	382.08%	90.81%	0.00%	−36.85%

**Table 7:** Goodness-of-fit statistics of the candidate probability distribution functions for the key parameters.

Parameter	Probability Density Function	*RMSE*	*R*
Partition coefficient, *K_d_* (L/mg)	Gumbel	0.0067	0.971
	Weibull	0.0019	0.998
	Frechet	0.1062	0.713
	Normal	0.0015	0.999
	Lognormal	0.0068	0.980
Settling velocity, *w_s_* (m/day)	Gumbel	0.18	0.967
	Weibull	0.11	0.988
	Frechet	0.26	0.947
	Normal	0.05	0.997
	Lognormal	0.08	0.994

## Data Availability

The raw data supporting the conclusions of this article will be made available by the authors upon request.

## Abbreviations

The following abbreviations are used in this manuscript:

APDC	Ammonium pyrrolidine dithiocarbamate
CFL	Courant–Friedrichs–Lewy
*ECI*	Estimation confidence interval
*MAE*	Mean absolute error
MCS	Monte Carlo simulation
MIBK	Methyl isobutyl ketone
Mn	Manganese
*NSE*	Nash–Sutcliffe efficiency
ORP	Oxidation–reduction potential
*RMS*	Root mean square
*RMSE*	Root mean square error
*RSR*	Ratio of the root mean square error to the standard deviation of the observations
SCHISM	Semi-implicit cross-scale hydroscience integrated system model
SCHISM-HM	Three-dimensional hydrodynamic–heavy metal transport model
*SS*	Skill score
*TSS*	Total suspended sediment
